# Effect of air pollution on clinical pregnancy outcomes following intrauterine insemination: a machine learning-based analysis

**DOI:** 10.3389/fpubh.2026.1931648

**Published:** 2026-09-18

**Authors:** Yiqing Guan, Liguang Huang, Jin Zhao, Yanfang Liang, Zehua Deng, Sichong Huang, Qiandong He, Yun Ye

**Affiliations:** Reproductive Center, Zhongshan City People’s Hospital, Zhongshan, China

**Keywords:** air pollution, carbon monoxide, clinical pregnancy, intrauterine insemination, machine learning, random forest model

## Abstract

**Objectives:**

Numerous studies have explored the impact of air pollution on *in vitro* fertilization outcomes; However, its effects on intrauterine insemination (IUI) remain unclear. This study investigated the association between air pollution exposure and clinical pregnancy outcomes following IUI.

**Methods:**

Clinical data from 2,328 IUI cycles performed at the Reproductive Centre of Zhongshan City People’s Hospital between January 2014 and December 2025 were retrospectively analysed together with air pollution data from the central urban area of Zhongshan during the same period. Data analysis in this study was conducted using machine learning algorithms and bioinformatics approaches.

**Results:**

Generalized estimating equation analysis showed that carbon monoxide (CO) exposure during the transition from the antral follicle stage to the mature follicle stage was significantly and inversely associated with clinical pregnancy. Least Absolute Shrinkage and Selection Operator regression analysis revealed a non-zero regression coefficient for CO. Random forest model analysis identified CO as one of the two most important predictors of clinical pregnancy during this stage. Furthermore, incorporation of air pollutant variables improved model performance, increasing precision, recall, F1-score, and the area under the precision–recall curve. Restricted cubic spline analysis demonstrated that the clinical pregnancy rate decreased from 16.09 to 8.50% with increasing CO concentrations during the same period. Gene Ontology and Kyoto Encyclopedia of Genes and Genomes enrichment analyses suggested that inflammation- and immune-related pathways may represent potential biological mechanisms underlying the observed association between CO exposure and reduced clinical pregnancy rates.

**Conclusion:**

Overall, these findings indicate that CO exposure during the transition from the antral follicle stage to the mature follicle stage is adversely associated with clinical pregnancy outcomes following IUI.

## 1 Introduction

Air pollution poses a threat to human reproductive health. In the general population, exposure to air pollution is associated with adverse pregnancy outcomes, including gestational hypertension (1, 2) and gestational diabetes (3, 4). Among individuals undergoing *in vitro* fertilization (IVF) treatment, air pollution may increase the risk of adverse outcomes, including reduced embryo implantation and live birth rates, as well as increased miscarriage and biochemical pregnancy rates (5–7). However, among individuals undergoing intrauterine insemination (IUI), the impact of air pollution on treatment outcomes remains unclear. Given the distinct pathophysiological and treatment protocols of IVF and IUI patients, the impact of air pollution on IVF outcomes cannot be directly applied to IUI. Thus, dedicated research into its effects on IUI outcomes is warranted.

The random forest model (RFM) is a widely used machine learning algorithm that is commonly employed for outcome prediction and variable selection in assisted reproductive treatment. Previous studies have shown that infertility duration, hormone levels on the day of human chorionic gonadotropin (HCG) administration, and sperm motility are significantly associated with live birth outcomes in assisted reproductive therapy, as identified using RFM (8). Another study reported that female age, body mass index (BMI), and baseline sex hormone levels were among the factors most strongly associated with clinical pregnancy following IUI (9). In addition, a study from Shanghai identified nitrogen dioxide (NO_2_) as one of the two most important factors influencing pregnancy outcomes after frozen embryo transfer (10). These findings suggest that RFM may be a useful tool for evaluating the potential impact of air pollutants on clinical pregnancy outcomes following IUI.

This study therefore aimed to investigate the effects of air pollutants on clinical pregnancy outcomes following IUI, using RFM in combination with generalized estimating equations (GEE) and restricted cubic splines (RCS). In addition, bioinformatics analyses were performed to identify human target genes interacting with air pollutants and to explore the associated biological processes and signaling pathways, thereby providing insights into the potential underlying mechanisms.

## 2 Methods

### 2.1 Study population

This single-centre retrospective study included couples who underwent IUI treatment cycles at the Reproductive Centre of Zhongshan City People’s Hospital between January 2014 and December 2025.

Inclusion criteria are as follows: (1) Couples diagnosed with infertility and eligible for IUI. (2) Undergoing IUI treatment. (3) The couples had long-term resided in the central urban area of Zhongshan.

Exclusion criteria included: (1) Infection with hepatitis C virus, human immunodeficiency virus, syphilis, gonorrhea, mycoplasma, or chlamydia. (2) Two IUI procedures performed in the same treatment cycle. (3) Artificial insemination with donor sperm. (4) Chromosomal abnormalities, prior surgeries or congenital anomalies of the urological or reproductive systems, long-term medication use, or exposure to toxins or radiation.

### 2.2 Air pollution exposure assessment

The central urban area of Zhongshan spans approximately 270 km^2^ and has a plain monsoon climate. Based on self-reported residential and workplace information, all couples included in this study were long-term residents of this central urban region, with both their residential and workplaces addresses located within the area before and during the IUI treatment cycle. Consequently, in their daily routines, they likely circulate across residential, commercial and recreational areas of the central urban area. There are four air quality monitoring stations in Zhongshan, including Huabai Station, Zhangxi Station, Zimaling Station and Nanqu Station. Geographically, these stations cover residential, commercial and recreational areas of the central urban area, matching the daily activity spaces of local residents. Following the methodology reported by Zeng et al. (11) for population exposure evaluation in plain based central urban areas, daily average pollutant concentrations from the four monitoring stations were used to quantify patients’ daily air pollution exposure.

Air pollutant concentration data were retrieved from the Environmental and Meteorological Data Services Platform,^1^ with raw observations sourced from official air quality monitoring stations under governmental administration. Daily concentrations of six criteria air pollutants (particulate matter with an aerodynamic diameter ≤2.5 μm [PM_2.5_], particulate matter with an aerodynamic diameter ≤10 μm [PM_10_], sulfur dioxide [SO_2_], NO_2_, CO, and ozone [O_3_]) were collected from air-monitoring stations throughout Zhongshan City for the period from June 2013 to December 2025. For missing data points, imputation was performed with the mean of the two nearest valid values within the preceding and subsequent 7 days.

Two exposure windows were defined based on the timing of IUI treatment to assess potential sensitive periods. Period 1 corresponded to the transition from the preantral to antral follicle stage and was defined as 90 to 15 days before the IUI procedure (approximately 76 days). Period 2 corresponded to the transition from the antral to mature follicle stage and was defined as 14 to 1 day before the IUI procedure (approximately 14 days). For each participant, the mean concentrations of the six air pollutants were calculated for both exposure windows.

### 2.3 Stimulation protocols

Female patients underwent either a natural cycle or an ovulation induction cycle for IUI treatment. Ovulation induction was achieved using clomiphene, letrozole, recombinant follicle-stimulating hormone (FSH), or human menopausal gonadotropin (HMG). Follicular development was monitored by ultrasonography. Once the dominant follicle reached maturity, 5,000–10,000 IU of HCG was administered intramuscularly, and IUI was performed 24–36 h later.

In some patients, a spontaneous luteinising hormone (LH) surge occurred when the mean follicular diameter reached 18 mm, resulting in natural ovulation without HCG administration. For these patients, IUI was performed either on the day of the LH surge or the following day.

### 2.4 Semen processing

Male patients were instructed to maintain sexual abstinence for 3–7 days before semen collection. Semen samples were collected by masturbation into a sterile container in the laboratory approximately 2 h before the IUI procedure. Following liquefaction, routine semen analysis was performed according to the *World Health Organization Laboratory Manual for the Examination and Processing of Human Semen (5th edition).*

Sperm preparation was performed using density-gradient centrifugation. Briefly, semen was carefully layered onto the density-gradient medium and centrifuged at 500 × g for 15 min. The supernatant was then discarded, and 4 mL of fertilization medium was added to resuspend the pellet. The sample was subsequently centrifuged at 300 × g for 5 min, after which the supernatant was discarded again. Finally, 0.5 mL of fertilization medium was added to resuspend the sperm, and sperm concentration and motility were assessed.

### 2.5 Intrauterine insemination procedure

IUI was performed using a soft catheter attached to a 1 mL syringe. Approximately 0.5–1 mL of the prepared sperm suspension was aspirated into the catheter and slowly injected into the uterine cavity, approximately 1–2 cm beyond the internal cervical os. After catheter removal, the patient remained in the supine position for 30 min.

### 2.6 Luteal phase support

Luteal phase support was individualized according to the clinical needs of each patient and was determined by the attending physician. Progestogen supplementation was initiated within 4 days after ovulation and was administered orally, intramuscularly, or vaginally. Oral dydrogesterone was prescribed at a dose of 20–40 mg/day, whereas intramuscular progesterone was administered at 20 mg/day. Alternatively, micronized progesterone was administered orally or vaginally at a dose of 200–400 mg/day.

After 14 days of luteal support, treatment was continued if pregnancy was confirmed by an HCG test and maintained until 35 days after ovulation. Following confirmation of an intrauterine pregnancy, the dose was gradually tapered and discontinued between 10 and 12 weeks of gestation.

### 2.7 Outcome measure

The primary outcome was clinical pregnancy. Clinical pregnancy was defined as a serum HCG level ≥30 U/L at 14 days after IUI, followed by ultrasonographic confirmation of a gestational sac within the uterine cavity on transvaginal ultrasound performed 4 weeks after IUI. Ectopic and biochemical pregnancies were not considered clinical pregnancies. The clinical pregnancy rate was calculated as follows: clinical pregnancy rate = (number of clinical pregnancy cases/number of IUI treatment cycles) × 100%.

### 2.8 Statistical analysis

All retrospective patient data were obtained from the electronic medical record system. Variables collected for analysis included female age, male age, stimulation protocol, BMI, basal FSH, basal LH, infertility duration, infertility type, luteal phase support, endometrial thickness, season, and calendar year, etc.

Continuous variables with a normal distribution were expressed as mean ± standard deviation and compared using the independent-samples *t*-test. Continuous variables with a non-normal distribution were expressed as median [interquartile range (IQR)] and compared using the independent-samples rank-sum test. Categorical variables were expressed as frequencies and percentages and compared using the chi-square test. Bivariate correlation analysis was conducted using the Spearman correlation coefficient. All statistical analyses were performed using SPSS version 26.0.

Multicollinearity among air pollutant exposure levels was assessed using the “car” package in R software by calculating variance inflation factor (VIFs). Variables with VIF values below 5 were retained. The relevant code is available in Supplementary Code 1.

GEE models were used to examine the associations between air pollutant exposure and clinical pregnancy outcomes following IUI. Air pollutant exposure was expressed as the IQR of the mean concentration during each exposure window. Associations between an IQR increase in pollutant concentration and clinical pregnancy outcomes were evaluated for both exposure windows. Results were reported as adjusted odds ratios with 95% confidence intervals. In this study, single-pollutant, two-pollutant, and multi-pollutant models were applied to evaluate the association between pollutants and clinical pregnancy outcomes. The assignment of independent variables in the GEE model is detailed in Supplementary Table S1.

Least Absolute Shrinkage and Selection Operator (LASSO) regression analysis was performed using the “glmnet” package in R for the sensitivity analysis of pollutants. During the analysis, covariates representing patients’ baseline characteristics were forced to be retained in the model, while a penalty was applied to the pollutant variables. The relevant code is provided in Supplementary Code 2.

The “randomForest” and “fastshap” packages in R software were used to construct RFMs and perform Shapley Additive exPlanations (SHAP) analyses, using the raw data. Variable importance was ranked according to the mean decrease in accuracy (MDA) and the mean absolute SHAP value (MASHAP). Grid search combined with 5-fold cross-validation was used to determine key hyperparameters in the model construction. Bootstrap internal validation and independent external validation were employed to assess model performance. The relevant code is provided in Supplementary Code 3. To evaluate the contribution of air pollutant variables to predictive performance, the RFM incorporating air pollutant variables was compared with the RFM excluding these variables. Given the class imbalance in the dataset, the Synthetic Minority Over-sampling Technique and Edited Nearest Neighbors (SMOTE-ENN) method was applied for data resampling before model reconstruction. The dataset was split at the patient level for modeling. After random shuffling, 70% of the patients were allocated to the training set and 30% to the testing set. The training data were processed using the SMOTE-ENN technique, whereas the testing data were kept intact. Model performance was assessed using precision, recall, F1-score, and the area under the precision–recall curve (AUPRC). These metrics were compared between the two models to evaluate the impact of incorporating air pollutant variables. The relevant code is provided in Supplementary Code 4.

Restricted cubic spline models were constructed using the “rms” package in R software to evaluate potential nonlinear associations between air pollutant concentrations and clinical pregnancy outcomes following IUI and to identify potential threshold or inflection points. The knot was set to 4. The relevant code is provided in Supplementary Code 5.

Bioinformatics analyses were conducted using publicly available databases. Human target genes interacting with air pollutants were obtained from the PubChem database (accessed 16 March 2026). Genes associated with female infertility were retrieved from the GeneCards database (accessed 16 March 2026) using the search term “female infertility.” For gene screening, the species was set to *Homo sapiens*, and the relevance score ≥5. The “tidyverse” and “ggvenn” packages in R software were used to identify overlapping genes and generate Venn diagrams. The relevant code is provided in Supplementary Code 6. The intersecting genes were subsequently subjected to Gene Ontology (GO) and Kyoto Encyclopedia of Genes and Genomes (KEGG) enrichment analyses using the Metascape website, the adjusted *p*-value was set to <0.05.

## 3 Results

### 3.1 Comparison of baseline characteristics and air pollutant exposure between groups

A total of 2,328 IUI cycles were included in this study. Following treatment, 318 cycles resulted in clinical pregnancy, whereas 2,010 cycles did not, yielding a clinical pregnancy rate of 13.66%. The study population was therefore divided into a clinical pregnancy group and a non-clinical pregnancy group.

No significant differences were observed between the groups in several baseline characteristics, including female age, male age, stimulation protocol, basal FSH level, luteal phase support, endometrial thickness, number of IUI cycles, and total progressive motile sperm count after semen processing (*p* > 0.05). However, significant differences were found in female BMI, basal LH level, basal FSH-to-LH ratio, infertility duration, and the number of follicles ≥14 mm on the day of IUI (*p* < 0.05; Table 1).

**Table 1:** Comparison of baseline characteristics between the clinical pregnancy and non-clinical pregnancy groups.

Variable	Clinical pregnancy (*N* = 318)	No clinical pregnancy (*N* = 2,010)	*p*-value
Female age (years)	31.05 ± 4.04	31.23 ± 4.30	0.631
Male age (years)	32.90 ± 4.69	33.12 ± 4.91	0.703
Female BMI (kg/m^2^)	22.01 ± 3.21	21.65 ± 3.22	0.036
Basal FSH (mIU/mL)	6.61 ± 2.43	6.67 ± 2.35	0.332
Basal LH (mIU/mL)	7.26 ± 5.64	6.50 ± 5.71	<0.001
Basal FSH-to-LH ratio	1.28 ± 0.87	1.40 ± 0.84	<0.001
Duration of infertility (years)	2.56 ± 1.75	2.77 ± 1.94	0.038
Endometrial thickness (mm)	10.20 ± 1.88	10.07 ± 1.80	0.295
Number of follicles ≥14 mm	1.24 ± 0.79	1.06 ± 0.63	<0.001
Total progressive motile sperm count after semen processing (×10^6^)	21.45 ± 12.00	21.02 ± 11.74	0.607
Number of IUI cycles	1.63 ± 0.86	1.68 ± 0.90	0.344
Stimulation protocol (*n*)			0.080
Natural cycle	137	972	
Ovulation-inducing cycle	181	1,038	
Type of infertility (*n*)			0.225
Primary	180	1,210	
Secondary	138	800	
Luteal phase support (*n*)			0.640
No	114	748	
Yes	204	1,262	

BMI, body mass index; FSH, follicle-stimulating hormone; IUI, intrauterine insemination; LH, luteinising hormone.

During Period 1, no significant differences were observed in exposure concentrations of PM_2.5_, PM_10_, or NO_2_ between the two groups (*p* > 0.05). However, exposure concentrations of SO_2_, CO, and O_3_ differed significantly between the groups (*p* < 0.05; Table 2).

**Table 2:** Comparison of air pollutant exposure between the clinical pregnancy and non-clinical pregnancy groups.

Pollutant	Clinical pregnancy (*N* = 318)	No clinical pregnancy (*N* = 2,010)	*p*-value
Period 1			
PM_2.5_ (μg/m^3^)	26.11 ± 12.17	26.85 ± 12.66	0.402
PM_10_ (μg/m^3^)	40.66 ± 15	41.94 ± 16.03	0.239
SO_2_ (μg/m^3^)	8.19 ± 5.52	8.64 ± 5.52	0.043
NO_2_ (μg/m^3^)	29.45 ± 12.99	30.29 ± 13.15	0.251
O_3_ (μg/m^3^)	85.05 ± 23.07	80.95 ± 23.36	0.010
CO (mg/m^3^)	0.76 ± 0.28	0.81 ± 0.30	0.011
Period 2			
PM_2.5_ (μg/m^3^)	26.55 ± 13.77	26.61 ± 15.00	0.665
PM_10_ (μg/m^3^)	41.57 ± 17.51	41.48 ± 19.27	0.500
SO_2_ (μg/m^3^)	7.88 ± 4.89	8.45 ± 6.00	0.215
NO_2_ (μg/m^3^)	30.16 ± 15.40	30.39 ± 16.04	0.995
O_3_ (μg/m^3^)	87.84 ± 34.93	81.75 ± 33.03	0.006
CO (mg/m^3^)	0.76 ± 0.29	0.80 ± 0.32	0.018

During Period 2, exposure concentrations of PM_2.5_, PM_10_, SO_2_, and NO_2_ did not differ significantly between the groups (*p* > 0.05). In contrast, exposure concentrations of CO and O_3_ showed significant between-group differences (*p* < 0.05; Table 2).

### 3.2 Multicollinearity analysis of air pollutants

Spearman correlation analysis of the six air pollutant variables in Periods 1 and Periods 2 revealed multiple coefficients with absolute values exceeding 0.6 (Supplementary Table S2), indicating strong inter-correlations among the pollutants. Subsequent variance inflation factor analysis identified PM_2.5_ and PM_10_ as the primary contributors to severe multicollinearity, with both exhibiting VIF values greater than 5 in various multipollutant models (Supplementary Table S3). Upon the simultaneous exclusion of PM_2.5_ and PM_10_, the VIF values for the remaining variables dropped below 5. This suggests that removing PM_2.5_ and PM_10_ effectively mitigates multicollinearity, thereby providing a more stable basis for accurately estimating the independent effects of SO_2_, NO_2_, CO, and O_3_. Additionally, the exposure distributions of the six air pollutants are summarized in Supplementary Table S4.

### 3.3 Association between air pollutant exposure and clinical pregnancy outcomes following IUI

GEE was employed to analyse the association between air pollutant exposure levels and IUI pregnancy outcomes. All models were adjusted for potential confounders, including female age, male age, female BMI, basal FSH-to-LH ratio, stimulation protocol, luteal phase support, endometrial thickness, infertility duration, infertility type, number of follicles ≥14 mm on the day of IUI, number of IUI cycles, total progressive motile sperm count after semen processing, season, and calendar year.

In the single-pollutant models for Period 2, no pollutants showed statistically significant associations (*p* > 0.05; Supplementary Table S6). However, the *p*-values for CO and O_3_ were close to the 0.05 threshold, suggesting potential effects on pregnancy outcomes. Consequently, two-pollutant models were further conducted for CO and O_3_ in Period 2. CO showed significant associations in some two-pollutant models (*p* < 0.05), whereas O_3_ remained no significant across all two-pollutant models (*p* > 0.05; Supplementary Table S6). Based on the multicollinearity analysis, we further constructed a multipollutant model using SO_2_, NO_2_, CO, and O_3_. The analysis showed that the effect estimate for CO remained robust and statistically significant (adjusted odds ratio = 0.709, 95% confidence interval: 0.543–0.927, *p* < 0.05), whereas O_3_ still showed no significant association (*p* > 0.05; Supplementary Table S6); In contrast, no significant associations were observed for any pollutants in the single-, two-, or multi-pollutant models for Period 1 (*p* > 0.05; Supplementary Table S5). Based on these findings, subsequent analyses will focus exclusively on Period 2, with a specific emphasis on the impact of CO exposure on IUI pregnancy outcomes.

### 3.4 Sensitivity analysis of pollutants based on the LASSO regression

To verify the robustness of the findings, LASSO regression model was further applied to the six air pollutant variables in Period 2. The results indicated that only CO and O_3_ retained non-zero regression coefficients (Supplementary Table S7), suggesting their relatively strong independent predictive value for IUI pregnancy outcomes after accounting for variable selection and multicollinearity. Consistent with the aforementioned multipollutant model results, CO demonstrated significant associations across multiple models, further confirming that CO exposure in Period 2 is a critical independent predictor of IUI pregnancy outcomes.

### 3.5 RFM analysis

A random forest model was applied to analyse the pollutant variables during Period 2. The model incorporating 14 baseline variables and four air pollutant variables (SO_2_, NO_2_, CO, and O_3_) from Period 2 was constructed to evaluate variable importance in predicting clinical pregnancy (Table 3). According to the MDA, the five most important variables were CO, NO_2_, SO_2_, calendar year, and season. Based on the MASHAP, the five most important variables were the basal FSH-to-LH ratio, CO, NO_2_, O_3_, and total progressive motile sperm count after semen processing. Both ranking methods consistently indicated that CO is one of the most critical predictors for clinical pregnancy outcomes (ranking within the top two), with NO_2_ also ranking within the top three. Consistent with the aforementioned multipollutant model and LASSO regression results, only CO consistently demonstrated superior predictive performance, this further confirms the robust predictive importance of CO in IUI pregnancy outcomes.

**Table 3:** Variable importance rankings in the random forest model.

Mean decrease in accuracy		Mean absolute SHAP value	
Rank	Variable	Rank	Variable
1	CO	1	Basal FSH-to-LH ratio
2	NO_2_	2	CO
3	SO_2_	3	NO_2_
4	Calendar year	4	O_3_
5	Season	5	Total progressive motile sperm count after semen processing

FSH, follicle-stimulating hormone; LH, luteinising hormone; SHAP, Shapley Additive exPlanations.

To evaluate the contribution of air pollutant variables to model performance, two RFMs were developed using the SMOTE-ENN resampling technique: one including air pollutant variables and the other excluding them. The model incorporating air pollutant variables demonstrated improved precision, recall, F1-score, and AUPRC compared with the model excluding these variables (Table 4). These findings suggest that air pollutant exposure contributes meaningfully to the prediction of clinical pregnancy outcomes following IUI and improves the predictive performance of the RFM.

**Table 4:** Comparison of random forest model performance with and without air pollutant variables.

Metrics	Model with air pollutant variables	Model without air pollutant variables
Precision	0.259	0.161
Recall	0.158	0.095
F1-score	0.196	0.119
AUPRC	0.171	0.153

AUPRC, area under the precision–recall curve.

### 3.6 Restricted cubic spline analysis

To ensure the scientific rigor of the study, nonlinear relationship analyses were restricted to CO during Period 2, as it was identified as a key predictor in the RFM, LASSO and GEE analyses. A restricted cubic spline model was constructed to evaluate the nonlinear association between CO exposure during Period 2 and clinical pregnancy outcomes following IUI. The model was adjusted for potential confounders, including female age, male age, female BMI, basal FSH-to-LH ratio, stimulation protocol, luteal phase support, endometrial thickness, infertility duration, infertility type, number of follicles ≥14 mm on the day of IUI, number of IUI cycles, total progressive motile sperm count after semen processing, calendar year, season, and exposure levels of SO_2_, NO_2_, and O_3_. RCS analysis did not reveal a statistically significant nonlinear dose–response relationship (*p* for nonlinearity >0.05) or overall association (*p* for overall >0.05) between CO exposure and IUI pregnancy outcomes. Consistent with the results from the GEE model, these findings suggest a significant linear association between CO exposure and IUI treatment outcomes. As shown in Figure 1, the clinical pregnancy rate decreased from 16.09 to 8.50% with increasing CO concentration.

**Figure 1:**
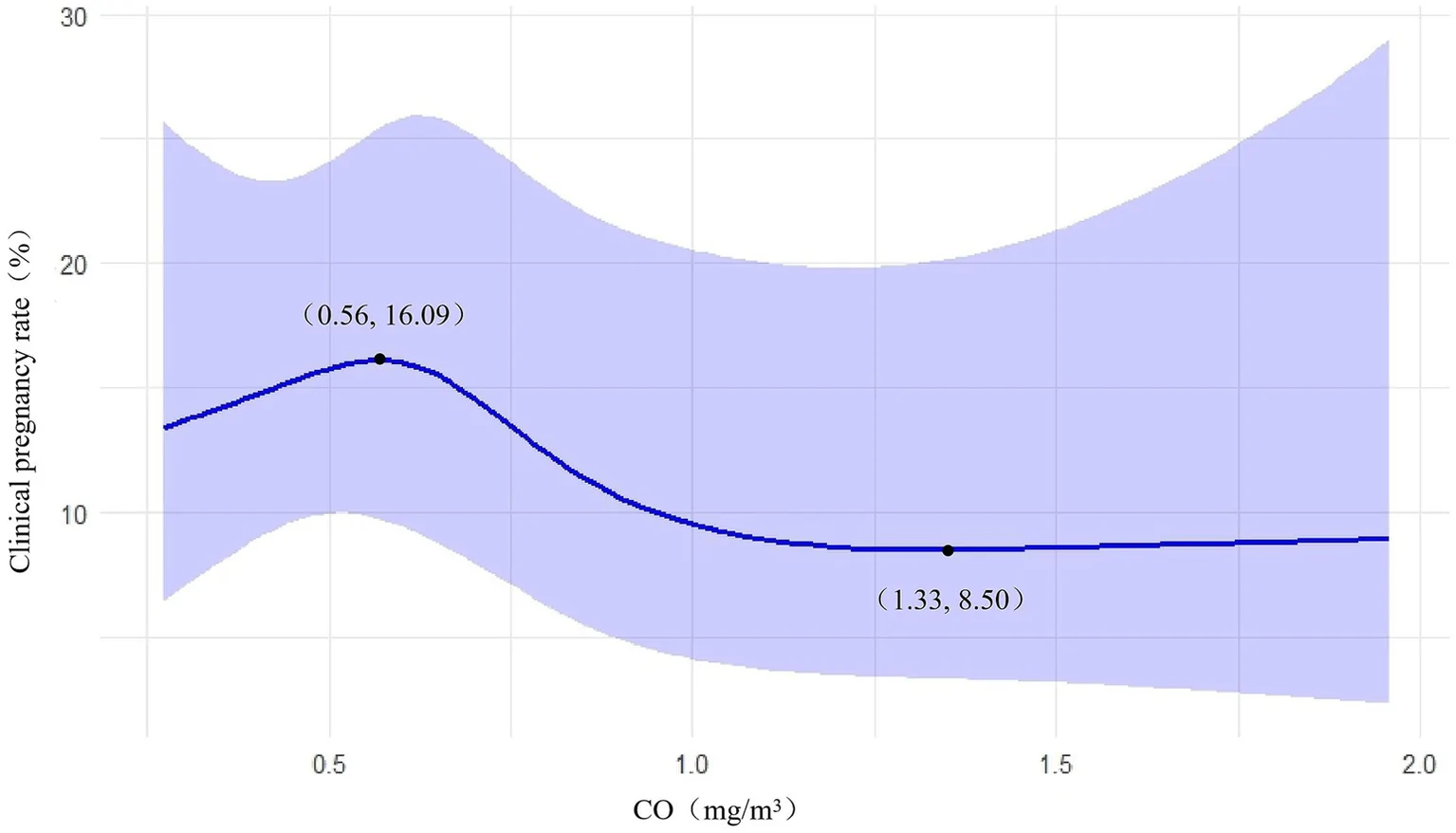
Restricted cubic spline analysis of the association between CO exposure and IUI clinical pregnancy rate.

### 3.7 Bioinformatics analysis

A total of 78 human target genes interacting with CO were retrieved from the PubChem database. A search for “female infertility” in the GeneCards database identified 2,308 associated genes. Comparison of the two gene sets revealed 34 overlapping genes (Figure 2).

**Figure 2:**
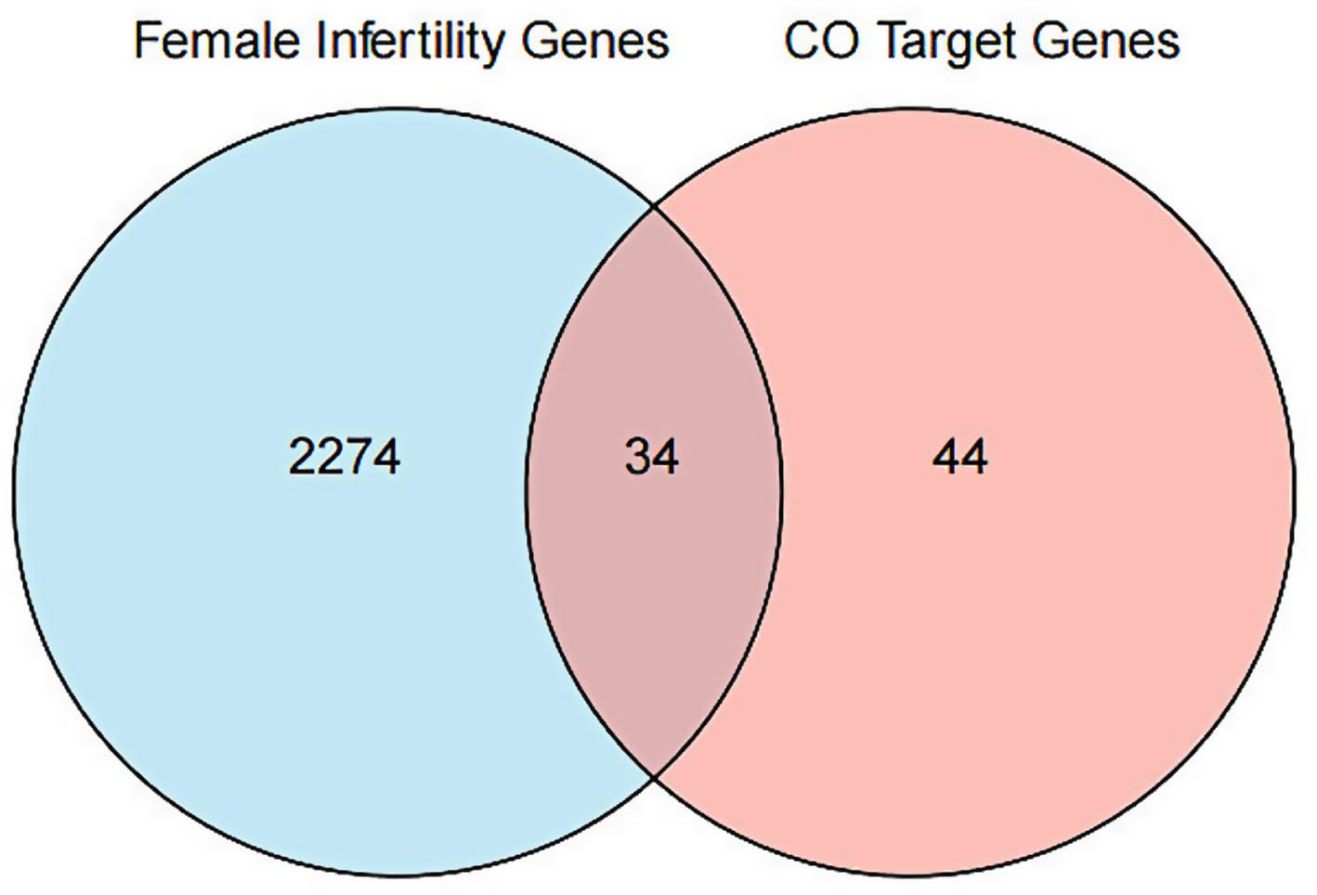
Venn diagram analysis showing the overlap between CO-interacting genes and female infertility-associated genes.

The overlapping genes were *IL6*, *TNF*, *PTEN*, *AKT1*, *IL10*, *SOD1*, *IFNG*, *FOXP3*, *CRP*, *CASP3*, *IL4*, *MIR21*, *CAT*, *HMOX1*, *HBB*, *IL18*, *XDH*, *ICAM1*, *RELA*, *MIR197*, *CASP9*, *FGB*, *MIR93*, *VCAM1*, *NFKBIA*, *CASP8*, *HBA2*, *MAPK14*, *SELP*, *COX7B*, *CYP2E1*, *MB*, *TNFRSF1B*, and *MIRLET7I*. These genes were subsequently submitted to the Metascape website for GO and KEGG enrichment analyses, the results of which are presented in Figures 3, 4.

**Figure 3:**
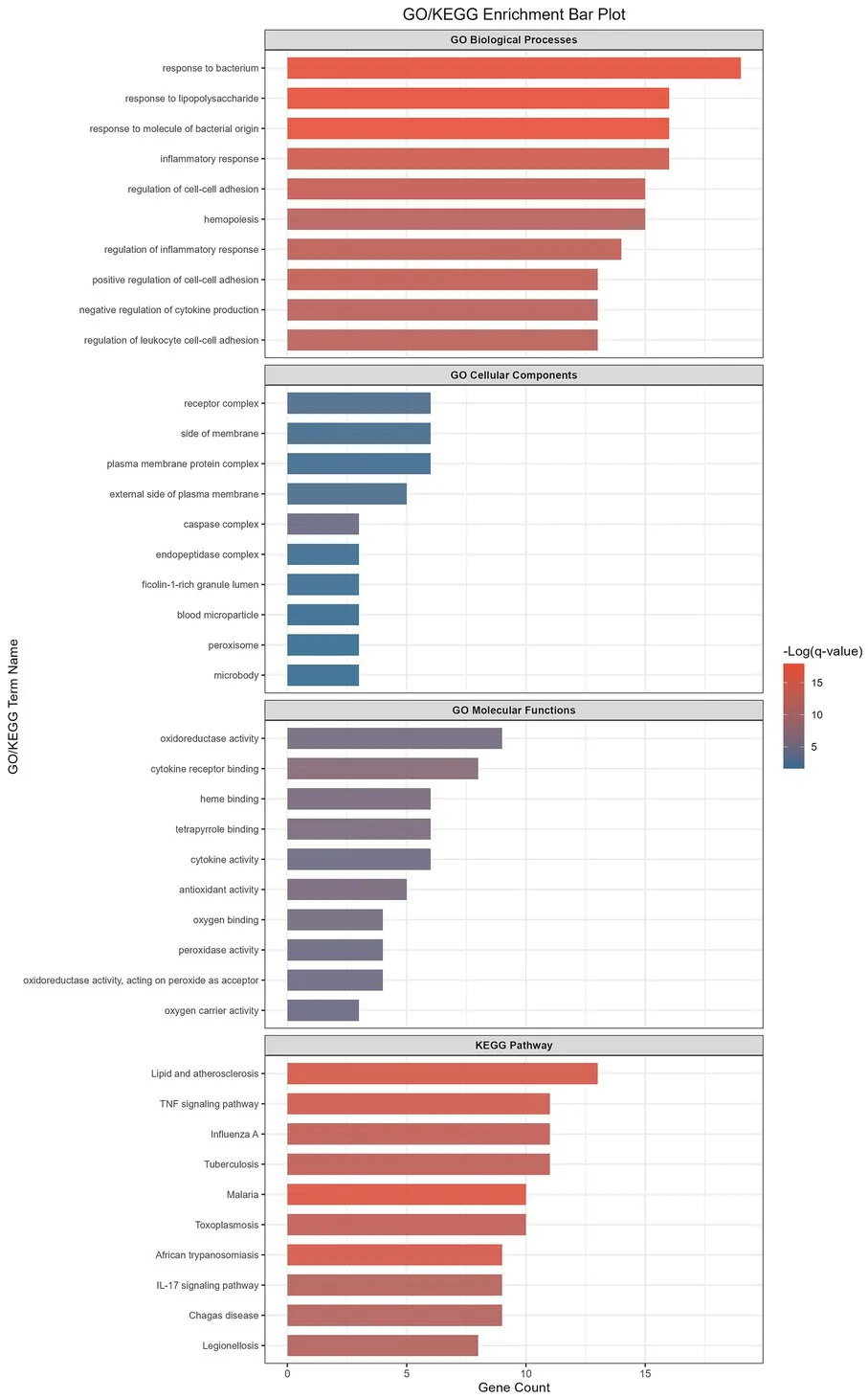
Gene Ontology (GO) and Kyoto Encyclopedia of Genes and Genomes (KEGG) enrichment analysis of overlapping genes presented as a bar chart.

**Figure 4:**
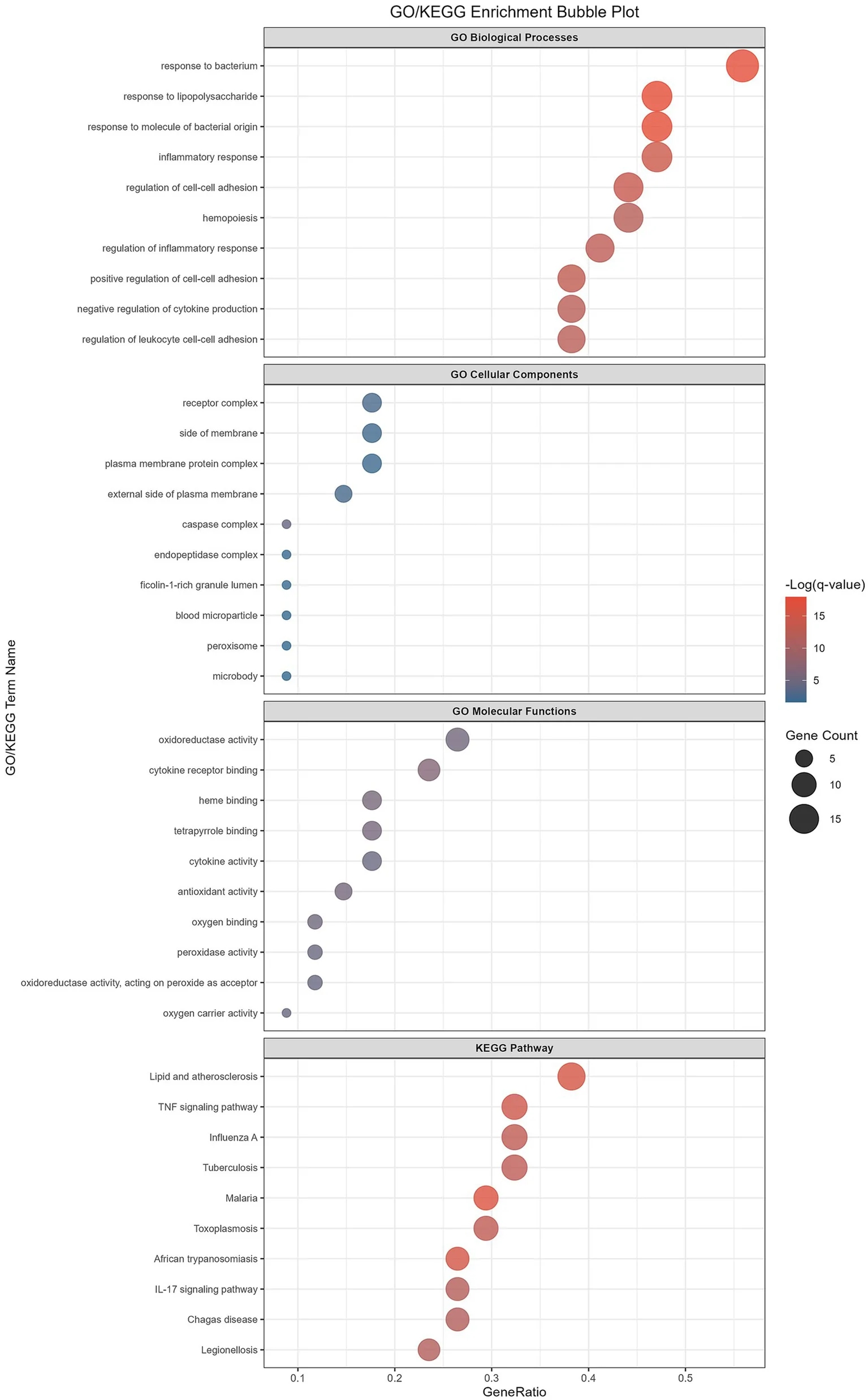
Gene Ontology (GO) and Kyoto Encyclopedia of Genes and Genomes (KEGG) enrichment analysis of overlapping genes presented as a bubble plot.

GO enrichment analysis indicates that these genes were primarily involved in biological processes related to responses to bacterial infection, inflammation, and immune regulation. KEGG pathway analysis showed significant enrichment in pathways associated with antimicrobial responses, inflammation, immune regulation, apoptosis, and atherosclerotic vascular lesions.

## 4 Discussion

### 4.1 Analysis of air pollutants

Air pollution has emerged as a major global public health challenge. Air pollutants comprise a complex mixture of gaseous pollutants, such as SO_2_, NO_2_, CO, and O_3_, as well as particulate pollutants, including particulate matter. In many urban environments, fine particulate matter is formed predominantly through secondary atmospheric processes involving gaseous precursors such as SO_2_, NO_2_, and CO. These processes include secondary oxidation, photochemical reactions, and organic aerosol transformation (12, 13). Consequently, the composition and concentration of urban air pollutants are strongly influenced by regional industrial activity, traffic density, and the effectiveness of environmental control measures.

In the present study, PM_2.5_ and PM_10_ were excluded through a variable-selection process. Following their removal, all remaining pollutants (SO_2_, NO_2_, CO, and O_3_) exhibited VIF values below 5, indicating the absence of significant multicollinearity. These findings suggest that PM_2.5_ and PM_10_ in the atmosphere of the central urban area of Zhongshan are closely linked to gaseous pollutants and may largely arise from secondary atmospheric transformation processes.

### 4.2 Analysis of sensitive exposure windows and potential mechanisms

To date, no studies have investigated the association between air pollutant exposure and IUI outcomes. Existing research on sensitive exposure windows has been limited to IVF treatment. Most studies have identified both the preantral-to-antral follicle stage and the antral-to-mature follicle stage as periods during which exposure to air pollutants may adversely affect reproductive outcomes. Li et al. (14) reported that increased concentrations of PM_2.5_, PM_10_, SO_2_, NO_2_, and CO during the preantral-to-antral follicle stage were significantly associated with lower clinical pregnancy rates following IVF. During the antral-to-mature follicle stage, however, only elevated SO_2_ and NO_2_ concentrations were associated with reduced clinical pregnancy rates. Liu et al. (15) found that exposure to PM_2.5_ and PM_10_ during either stage did not affect implantation, clinical pregnancy, or live birth rates. Nevertheless, exposure during the preantral-to-antral follicle stage was associated with reduced oocyte maturation and fertilization rates, whereas exposure during the antral-to-mature follicle stage was associated with significant reductions in oocyte maturation, fertilization, cleavage, and high-quality embryo rates. Similarly, Deng et al. (16) reported significant dose-dependent inverse associations between exposure to PM_2.5_, SO_2_, NO_2_, CO, and O_3_ during the preantral-to-antral follicle stage and both total oocyte counts and mature oocyte counts. During the antral-to-mature follicle stage, only NO_2_ and O_3_ exposure were significantly associated with reductions in total oocyte numbers.

For the first time, this study investigated the association between air pollutant exposure and IUI outcomes. Single-pollutant, two-pollutant, and multi-pollutant models analyses revealed no significant association between air pollutant exposure during the preantral-to-antral follicle stage and clinical pregnancy outcomes. In contrast, CO exposure during the antral-to-mature follicle stage was significantly and inversely associated with clinical pregnancy. Unlike findings reported for IVF, IUI outcomes did not appear to be sensitive to air pollutant exposure during the preantral-to-antral follicle stage. Several factors may explain this difference. First, the clinical pregnancy rate following IUI is typically approximately 15%, compared with 40–60% following IVF. The substantially lower success rate of IUI may reduce the ability to detect the adverse effects of air pollutant exposure. Second, compared with patients undergoing IVF, those receiving IUI generally have better ovarian reserve and less severe reproductive impairment, which may enhance their capacity to compensate for or repair damage associated with environmental exposures.

The observed sensitivity during the antral-to-mature follicle stage may also have several explanations. First, this stage is characterized by rapid expansion of the follicular antrum, increased follicular fluid volume, and enhanced perifollicular vascularization and blood flow. These physiological changes may facilitate the passage of air pollutants across the blood–follicle barrier and promote their accumulation within follicular fluid, thereby increasing the risk of oocyte damage (17). Second, the antral-to-mature follicle stage is considerably shorter than the preantral-to-antral follicle stage. Consequently, oocytes may have insufficient time to repair pollutant-induced damage before ovulation and fertilization, potentially reducing the likelihood of successful pregnancy. Third, the endometrium undergoes active proliferation during the antral-to-mature follicle stage. Air pollutant exposure during this period may therefore impair endometrial receptivity and adversely affect implantation and subsequent pregnancy outcomes (18).

### 4.3 RFM analysis

The random forest model, a widely used machine learning approach, consists of multiple decision trees, each of which independently generates a prediction. The final classification is determined through aggregation of the individual tree predictions, typically using a majority-voting mechanism. This ensemble approach enables numerous weak classifiers to be combined into a stronger and more robust predictive model (10, 19, 20).

When developing classification models, environmental factors such as light, weather conditions, noise, and air pollution are often regarded as “weak classifiers” because of their relatively low predictive accuracy, instability, simple structure, and strong complementarity with other variables (10, 19, 20). Consequently, environmental exposure variables are particularly well suited for inclusion in RFMs. In addition, the clinical pregnancy rate following IUI is approximately 15%, whereas the treatment failure rate approaches 85%, resulting in a highly imbalanced dataset. Previous studies have demonstrated that RFMs can effectively address challenges associated with imbalanced data, particularly when combined with appropriate resampling techniques (21, 22). Therefore, the RFM represents a suitable analytical and predictive approach for evaluating the contribution of air pollutant exposure to clinical pregnancy outcomes in the present study. In this study, air pollutant variables were incorporated into the RFM. Both feature-importance ranking methods identified CO as one of the two most important predictors of clinical pregnancy following IUI. This finding was consistent with the results of the GEE and LASSO analysis.

The classification of imbalanced datasets remains one of the most challenging problems in machine learning. When substantial class imbalance exists, classification performance may be adversely affected because models tend to favor the majority class. As a result, overall accuracy may appear high, while predictive performance for the minority class remains poor (23, 24). In the present study, the clinical pregnancy rate following IUI was approximately 15%, making accurate prediction of successful pregnancies particularly important. A common approach for addressing class imbalance is resampling, which seeks to balance class distributions by generating, removing, or modifying data samples. In this study, the SMOTE-ENN resampling method was employed because of its reported advantages in improving both predictive performance and model sensitivity (25, 26). In addition, conventional performance metrics, such as the receiver operating characteristic curve, the area under the receiver operating characteristic curve, and overall accuracy, have recognized limitations when applied to imbalanced datasets (27, 28). Given the focus on predicting the minority class, model performance was evaluated using metrics that are more informative in this setting, namely precision, recall, F1-score, and AUPRC (28). Using these approaches, the RFM incorporating air pollutant variables demonstrated improved precision, recall, F1-score, and AUPRC compared with the model excluding these variables. These findings are consistent with previous reports by Wan et al. (10) and Aw et al. (29), suggesting that environmental factors may contribute meaningfully to classification performance but are most informative when considered alongside other clinical and demographic variables.

### 4.4 Bioinformatics analysis of the potential mechanisms linking air pollutant exposure to reproductive outcomes

As a major component of air pollution, CO exerts complex physiological effects on human health. Endogenous CO is generated through the oxidative degradation of haem by haem oxygenase (30). Previous studies have shown that endogenous CO functions similarly to nitric oxide (NO) as a gaseous signaling molecule (31, 32) and plays important physiological roles in the nervous (33, 34) and cardiovascular systems (35). Within the female reproductive system, direct evidence demonstrating a regulatory role for CO in reproductive physiology remains limited. Nevertheless, haem oxygenase activity has been identified in the ovary (36), uterus (37), and placental tissue (38), suggesting that CO is produced locally within reproductive tissues and may exert important physiological functions. Němeček et al. proposed that physiological concentrations of CO may inhibit excessive NO production, thereby suppressing activation of the NO-mediated soluble guanylate cyclase/cyclic guanosine monophosphate signaling pathway. This mechanism may inhibit ovarian steroidogenesis and the first meiotic division of oocytes (39). In addition, Bilban et al. suggested that CO may bind to mitochondrial cytochrome c oxidase, resulting in the generation of reactive oxygen species. This transient increase in reactive oxygen species may upregulate p38 signaling and subsequently inhibit the second meiotic division of oocytes (40, 41).

Whether chronic, low-dose exposure to exogenous CO affects these physiological processes remains an important question. CO exposure causes systemic tissue hypoxia, thereby inducing systemic oxidative stress and vascular endothelial injury (42, 43), which may reduce the likelihood of clinical pregnancy. Epidemiological studies have consistently shown that long-term exposure to exogenous CO is associated with reduced fertility and adverse pregnancy outcomes in women (44–46). Consistent with these findings, the present study demonstrated that chronic exposure to exogenous CO was associated with a reduced clinical pregnancy rate following IUI. Nevertheless, the aforementioned studies only demonstrated a correlation between CO exposure and female infertility and cannot directly establish a causal relationship. CO may merely serve as a proxy for other pollutants to reflect the observed effects. Nevertheless, as a substance exerting systemic toxic effects on humans, investigating the toxicological mechanisms of CO itself on female reproduction still helps provide certain biological plausibility for this epidemiological association. On the other hand, even if combined exposure to CO and other pollutants induces synergistic toxicity, elucidating the potential reproductive toxicological mechanisms of CO remains critical to understanding female reproductive health under complex environmental exposure. However, the biological mechanisms through which chronic CO exposure impairs female fertility remain poorly understood. To address this knowledge gap, we employed a bioinformatics approach to explore potential mechanisms underlying CO-induced reproductive impairment.

Bioinformatics analysis identified 78 human genes that interact with CO, of which 34 were also associated with female infertility. GO enrichment analysis of these overlapping genes revealed significant enrichment of biological processes related to antimicrobial responses, including response to lipopolysaccharide, response to molecules of bacterial origin, and response to bacteria. The remaining enriched biological processes were primarily related to inflammation and immune regulation, including inflammatory response, regulation of cell–cell adhesion, positive regulation of cell–cell adhesion, regulation of inflammatory response, negative regulation of cytokine production, regulation of leukocyte cell–cell adhesion, and hemopoiesis. Similarly, KEGG pathway analysis demonstrated enrichment of several pathways related to host defense against microbial infection, including malaria, African trypanosomiasis, toxoplasmosis, influenza A, tuberculosis, legionellosis, and Chagas disease. In addition, the tumor necrosis factor and interleukin-17 signaling pathways, both of which play key roles in inflammatory and immune responses, were among the most significantly enriched pathways. Collectively, these findings suggest that CO exposure may promote inflammatory and immune responses within the female reproductive system. Localized low-grade ovarian inflammation may impair follicular development and reduce oocyte quality, ultimately compromising embryo quality and pregnancy outcomes (47). Likewise, inflammation within the uterine environment may enhance immune-mediated rejection of the embryo and reduce implantation success (48). Therefore, inflammation and immune dysregulation may represent key mechanisms linking CO exposure to reduced clinical pregnancy rates following IUI. KEGG analysis also identified enrichment of the lipid and atherosclerosis pathway, suggesting a potential role for vascular dysfunction. One possible explanation is that CO exposure promotes localized atherosclerotic changes within ovarian and uterine vasculature, facilitating immune cell recruitment and infiltration into reproductive tissues and thereby contributing to chronic low-grade inflammation (49, 50). Furthermore, enrichment of the tumor necrosis factor signaling pathway suggests a potential role for apoptosis in mediating reproductive damage. Increased apoptotic activity may promote oocyte degeneration and reduce embryo quality (51). Taken together, these findings suggest that inflammation, immune dysregulation, vascular injury, and apoptosis may collectively contribute to the adverse effects of chronic CO exposure on clinical pregnancy outcomes following IUI.

### 4.5 Clinical implications

The present study identified a significant inverse association between CO exposure and clinical pregnancy outcomes following IUI in GEE. Restricted cubic spline analysis further demonstrated a marked decline in the clinical pregnancy rate. Given that vehicular exhaust is the primary source of CO in the central urban area of Zhongshan, we recommend that female patients undergoing IUI treatment minimize their exposure to traffic-related pollution during the treatment to mitigate potential reproductive health risks. In addition, monitoring ambient air pollution levels may provide useful context when interpreting temporal fluctuations in IUI success rates. If an unexplained decline in clinical pregnancy rates is observed, concurrent environmental exposure data may help determine whether air pollution represents a contributing factor.

### 4.6 Limitations

This study has several limitations. First, the retrospective single-centre design may have introduced selection bias and limited the generalizability of the findings. Second, the proposed mechanisms underlying the effects of CO exposure were derived from bioinformatics analyses and require validation through experimental and mechanistic studies. Third, CO data were derived from fixed air monitoring stations, which may serve as a proxy for other unmeasured pollution sources and fails to capture individual exposure variations arising from smoking, occupation, socioeconomic status, and indoor exposure, leading to exposure misclassification and residual confounding bias. Fourth, meteorological factors such as temperature and humidity were not included in the current analysis, which may also introduce residual confounding bias. Finally, the dataset was not partitioned chronologically. This non-temporal splitting may introduce temporal leakage, potentially affecting the robustness of the analytical results.

## 5 Conclusion

In this retrospective study, we investigated the association between air pollution exposure and clinical pregnancy outcomes following IUI treatment. The findings demonstrated that CO exposure during the transition from the antral follicle stage to the mature follicle stage was significantly and inversely associated with clinical pregnancy. Furthermore, bioinformatics analysis provided potential biological insights into this association, suggesting that inflammation- related and immune-related pathways may underlie the observed reproductive impairment. These findings highlight the need for further experimental validation to clarify the causal mechanisms.

## Data Availability

The datasets presented in this article are not readily available because ethical and legal reasons. Requests to access the datasets should be directed at lrhmail16@163.com.

## Footnotes

1 http://eia-data.com.
