# Supplementary material for: Effect of air pollution on clinical pregnancy outcomes following intrauterine insemination: a machine learning-based analysis
Source: Front Public Health. 2026 Sep 18;14:1931648. doi: 10.3389/fpubh.2026.1931648 (PMC13630761; doi:10.3389/fpubh.2026.1931648)
Supplement: Supplementary file 1 [file Supplementary_file_1.docx]

**Code 1: Code for calculating Variance Inflation Factor**

library(car)	# Core package: compute variance inflation factor using vif()

\# --- Configuration ---

\# Set CSV file path (modify to full path if not in the current working directory)

\# Set working directory to where the dataset is stored

setwd("C:\\Users\\R\\Desktop\\R\\R")

\# 1. Read CSV file (ensure the file path is correct; use file name directly if it is under the working directory)

P2 <- read.csv("P2.csv")

\# 2. Extract data from column 3 to column 7

data_vars <- P2[, 3:7]

\# 3. Remove rows with missing values (missing values are not permitted for VIF calculation)

data_vars <- na.omit(data_vars)

\# 4. Generate random dependent variable (VIF is determined solely by multicollinearity among independent variables and is independent of the dependent variable)

set.seed(123) # Fix random seed to ensure reproducible results

y <- rnorm(nrow(data_vars))

\# 5. Fit linear model with all target columns as independent variables

lm_model <- lm(y ~ ., data = data_vars)

\# 6. Calculate and output variance inflation factor

vif_result <- vif(lm_model)

print(vif_result)

**Code 2: Code for LASSO Analysis**

library(glmnet) # Core package for LASSO analysis

library(dplyr)	# Data processing package

library(pROC)	# Package for AUC calculation and plotting

\# Set working directory to the location of the dataset

setwd("C:\\Users\\R\\Desktop\\R\\R")

\# ============= 2. Read Data + Force Validation of Data Structure =====================

data <- read.csv("P2.csv", stringsAsFactors = FALSE)

\# ------------ Critical Validation: Ensure data structure meets requirements ------------

\# 1. Check total number of columns (at least 23 columns; outcome variable in column 23)

if (ncol(data) < 23) {

stop("Error: The CSV file contains fewer than 23 columns. Please check the file structure!")

}

\# 2. Outcome variable: Column 23 is the outcome, 0 = non‑pregnancy, 1 = pregnancy

y_raw <- data[,23]

y_no_na <- y_raw[!is.na(y_raw)]

if (!all(y_no_na %in% c(0, 1))) {

stop("Error: Outcome variable in column 23 must be binary numeric (0/1)! 0 = non‑pregnancy, 1 = pregnancy")

}

\# Print basic dataset information for verification

cat("===== Basic Dataset Information =====\n")

cat("Total sample size: ", nrow(data), "\n")

cat("Total number of columns: ", ncol(data), "\n")

cat("Distribution of outcome variable (column 23):\n", table(y_raw), "\n")

cat("Range of independent variables: Column 3 ~ Column 22\n")

cat("Penalized variables: Column 3 ~ Column 8\n")

cat("Forced‑retained variables: Column 9 ~ Column 22\n")

\# ===================== 3. Data Preprocessing =====================

\# 3.1 Independent variables: Column 3 ~ Column 22; columns 21 and 22 have been manually dummy‑coded

X_raw <- data[, 3:22]

\# 3.2 Set penalty factor

\# X_raw has 20 columns in total:

\# Columns 1‑6 of X_raw → original columns 3‑8: penalty = 1

\# Columns 7‑20 of X_raw → original columns 9‑22: penalty = 0 (forced retention)

penalty.factor <- c(rep(1, 6), rep(0, 14))

\# Validate that the length of penalty factor matches the number of independent‑variable columns

if (length(penalty.factor) != ncol(X_raw)) {

stop(paste0("Error: Length of penalty factor (", length(penalty.factor), ") does not match number of independent‑variable columns (", ncol(X_raw), ")!"))

}

\# 3.3 Missing value handling

cat("\n===== Missing Value Check =====\n")

missing_per_col <- colSums(is.na(X_raw))

print(missing_per_col)

cat("Total rows with missing values: ", sum(complete.cases(X_raw, y_raw) == FALSE), "\n")

\# Remove rows containing missing values

complete_idx <- complete.cases(X_raw, y_raw)

y_clean <- y_raw[complete_idx]

X_clean <- as.matrix(X_raw[complete_idx, ])

cat("\nValid sample size after cleaning: ", length(y_clean), "\n")

cat("Number of independent‑variable columns after cleaning: ", ncol(X_clean), "\n")

\# ===================== 4. LASSO Model Construction =====================

set.seed(13924) # Fix random seed for reproducible results

cv_lasso <- cv.glmnet(

x = X_clean,

y = y_clean,

family = "binomial",

penalty.factor = penalty.factor,

nfolds = 10,

type.measure = "auc",

trace.it = 1

)

\# 4.2 Inspect cross‑validation results

cat("\n===== Cross‑Validation Results =====\n")

plot(cv_lasso)

cat("Optimal lambda value (maximum AUC): ", cv_lasso$lambda.min, "\n")

cat("Lambda value under 1‑SE criterion (more parsimonious model): ", cv_lasso$lambda.1se, "\n")

\# 4.3 Final LASSO model (lambda.min)

final_lasso <- glmnet(

x = X_clean,

y = y_clean,

family = "binomial",

penalty.factor = penalty.factor,

lambda = cv_lasso$lambda.min

)

\# ===================== 5. Result Interpretation and Output =====================

lasso_coef <- coef(final_lasso)

selected_vars <- rownames(lasso_coef)[which(lasso_coef != 0)]

cat("\n===== Variables Selected in Final Model =====\n")

print(selected_vars)

\# Regression coefficients and odds ratios (OR)

coef_df <- data.frame(

Variable = rownames(lasso_coef),

Regression_coefficient = as.numeric(lasso_coef),

OR = exp(as.numeric(lasso_coef)),

Row_number = 1:nrow(lasso_coef)

)

coef_df <- coef_df %>%

filter(Variable != "(Intercept)") %>%

arrange(desc(OR))

cat("\n===== Coefficients and OR Values of Final Model =====\n")

print(coef_df, row.names = FALSE)

\# Model prediction and evaluation

pred_prob <- predict(final_lasso, newx = X_clean, type = "response")

pred_class <- ifelse(pred_prob > 0.5, 1, 0)

conf_matrix <- table(True_value = y_clean, Predicted_value = pred_class)

cat("\n===== Training‑Set Model Evaluation =====\n")

cat("Confusion matrix:\n")

print(conf_matrix)

train_auc <- auc(roc(y_clean, pred_prob))

cat("Training‑set AUC: ", round(train_auc, 4), "\n")

\# ===================== 6. Save Results =====================

write.csv(coef_df, "LASSO_model_coefficient_results.csv", row.names = FALSE)

saveRDS(final_lasso, "LASSO_final_model.rds")

saveRDS(cv_lasso, "LASSO_crossvalidation_results.rds")

**Code 3: Code for Random Forest and SHAP‑based Feature Importance Ranking Analysis**

\# Load core packages

library(randomForest)	# Random forest core package

library(parallelly)

library(caret)	# Confusion matrix, data partitioning

library(pROC)	# AUC calculation and confidence intervals

library(ResourceSelection) # Hosmer-Lemeshow test

library(dplyr)	# Data manipulation

library(ggplot2)	# Visualization

library(PRROC)	# PR curve and AUC-PR

library(boot)	# Bootstrap confidence intervals

library(fastshap)	# Stable SHAP analysis package

library(tidyr)	# Data reshaping

setwd("C:\\Users\\R\\Desktop\\R\\R"); # Working directory for data

set.seed(123)

\# 1. Data import and preprocessing

data <- read.csv("TP22.csv", fileEncoding = "UTF-8", stringsAsFactors = FALSE)

str(data) # Inspect data structure

\# Convert specified columns to factors

factor_columns <- c(4, 8, 12, 13, 14) # Define column indices to be converted to factors

for (col in factor_columns) {

data[, col] <- as.factor(data[, col])

}

\# ==== Critical correction: Recode outcome as neg/pos to avoid errors with numeric levels =======

data[, 19] <- factor(data[, 19], levels = c(0,1), labels = c("neg","pos"))

colnames(data)[19] <- "outcome" # Rename outcome column

\# Data split: 70% training set, 30% test set (fixed independent external test set, unchanged throughout)

train_index <- createDataPartition(data$outcome, p = 0.7, list = FALSE)

train_data <- data[train_index, ]

test_data <- data[-train_index, ]

#==================== Custom AUC-PR evaluation function ====================

prSummary <- function(data, lev = NULL, model = NULL) {

pos_level <- lev[2]

prob <- data[, pos_level]

label <- ifelse(data$obs == pos_level, 1, 0)

if(length(unique(label)) < 2){

out <- c(AUCPR = NA)

}else{

pr <- pr.curve(scores.class0 = prob[label == 1], scores.class1 = prob[label == 0], curve = FALSE)

out <- c(AUCPR = pr$auc.integral)

}

names(out) <- "AUCPR"

out

}

#===== Custom random forest trainer supporting simultaneous tuning of mtry + nodesize ===

rf_custom <- list(

label = "randomForest_custom",

library = "randomForest",

type = "Classification",

parameters = data.frame(

parameter = c("mtry", "nodesize"),

class = c("numeric", "numeric"),

label = c("mtry", "nodesize")

),

grid = function(x, y, len = NULL, search = "grid") {

expand.grid(mtry = seq(2,8,1), nodesize = c(5,10,15,20))

},

fit = function(x, y, wts, param, lev, last, classProbs, ...) {

randomForest(x = x, y = y,

mtry = param$mtry,

nodesize = param$nodesize,

ntree = 1000,

maxnodes = 1000,

importance = TRUE)

},

predict = function(modelFit, newdata, preProc = NULL, submodels = NULL) {

predict(modelFit, newdata)

},

prob = function(modelFit, newdata, preProc = NULL, submodels = NULL) {

predict(modelFit, newdata, type = "prob")

},

varImp = function(object, ...) {

imp <- importance(object)

out <- data.frame(Overall = imp[,1])

rownames(out) <- rownames(imp)

out

},

levels = function(x) x$classes

)

\# Cross-validation settings

ctrl_tune <- trainControl(

method = "cv",

number = 5,

summaryFunction = prSummary,

classProbs = TRUE,

verboseIter = FALSE

)

\# Hyperparameter grid for tuning

rf_grid <- expand.grid(

mtry = seq(4, 8, by = 1),

nodesize = c(10, 15, 20)

)

cat("\n= Start hyperparameter grid tuning (optimize to maximize AUC-PR, mtry + nodesize) ===\n")

set.seed(123)

rf_tune <- train(

x = train_data[, !colnames(train_data) %in% "outcome"],

y = train_data$outcome,

method = rf_custom,

tuneGrid = rf_grid,

trControl = ctrl_tune,

metric = "AUCPR",

maximize = TRUE

)

\# Output optimal parameters

best_mtry <- rf_tune$bestTune$mtry

best_nodesize <- rf_tune$bestTune$nodesize

cat("Optimal mtry =", best_mtry, "\n")

cat("Optimal nodesize =", best_nodesize, "\n")

\# Train final model with optimal hyperparameters

rf_model <- randomForest(

outcome ~ .,

data = train_data,

ntree = 1000,

mtry = best_mtry,

nodesize = best_nodesize,

maxnodes = 1000,

importance = TRUE,

na.action = na.omit

)

#=============== Bootstrap internal validation (200 resamples) ====================

boot_iter <- 200

boot_metrics <- data.frame(

AUCROC = numeric(boot_iter),

AUCPR = numeric(boot_iter)

)

cat("\n===== Start Bootstrap internal validation (", boot_iter, " resamplings) =====\n")

set.seed(123)

for(b in 1:boot_iter){

boot_idx <- sample(1:nrow(train_data), size = nrow(train_data), replace = TRUE)

boot_train <- train_data[boot_idx, ]

boot_model <- randomForest(

outcome ~ .,

data = boot_train,

ntree = 1000,

mtry = best_mtry,

nodesize = best_nodesize,

maxnodes = 1000,

importance = FALSE,

na.action = na.omit

)

boot_prob <- predict(boot_model, train_data, type = "prob")[, "pos"]

lab <- ifelse(train_data$outcome == "pos", 1, 0)

roc_tmp <- roc(lab, boot_prob, quiet = TRUE)

boot_metrics$AUCROC[b] <- auc(roc_tmp)

pr_tmp <- pr.curve(scores.class0 = boot_prob[lab==1], scores.class1 = boot_prob[lab==0], curve=FALSE)

boot_metrics$AUCPR[b] <- pr_tmp$auc.integral

if(b %% 50 == 0) cat("Completed bootstrap iteration: ", b, "/", boot_iter, "\n")

}

cat("\n===== Summary of Bootstrap internal validation results =====\n")

print(summary(boot_metrics))

cat("Bootstrap mean AUC-ROC =", round(mean(boot_metrics$AUCROC),4),

"95%CI:", round(quantile(boot_metrics$AUCROC,0.025),4), "~", round(quantile(boot_metrics$AUCROC,0.975),4),"\n")

cat("Bootstrap mean AUC-PR =", round(mean(boot_metrics$AUCPR),4),

"95%CI:", round(quantile(boot_metrics$AUCPR,0.025),4), "~", round(quantile(boot_metrics$AUCPR,0.975),4),"\n")

#==================== Variable importance ====================

imp_accuracy <- importance(rf_model, type = 1)

imp_accuracy_df <- data.frame(

Parameter = rownames(imp_accuracy),

Importance = imp_accuracy[, 1],

row.names = NULL

) %>% arrange(desc(Importance))

cat("=== Mean Decrease in Accuracy ===\n")

print(imp_accuracy_df)

varImpPlot(rf_model, main = "Traditional variable importance of random forest")

#==================== Confusion matrix ====================

train_pred <- predict(rf_model, train_data)

train_cm <- confusionMatrix(train_pred, train_data$outcome, positive = "pos")

cat("\n=== Training set confusion matrix ===\n")

print(train_cm)

test_pred <- predict(rf_model, test_data)

test_cm <- confusionMatrix(test_pred, test_data$outcome, positive = "pos")

cat("\n=== Test set confusion matrix ===\n")

print(test_cm)

#=========== Function for bootstrap confidence interval of PR-AUC ====================

calc_pr_auc <- function(data, indices) {

prob <- data$prob[indices]

label <- data$label[indices]

if (length(unique(label)) < 2) return(NA)

if (length(unique(prob)) == 1) return(0.5)

pr_obj <- tryCatch({

pr.curve(scores.class0 = prob[label == 1], scores.class1 = prob[label == 0], curve = FALSE)

}, error = function(e) { return(list(auc.integral = NA)) })

return(pr_obj$auc.integral)

}

get_pr_auc_ci <- function(prob_vec, label_vec, seed = 123, R = 1000) {

set.seed(seed)

pr_data <- data.frame(prob = prob_vec, label = label_vec)

boot_result <- boot(data = pr_data, statistic = calc_pr_auc, R = R)

boot_result$t <- boot_result$t[!is.na(boot_result$t)]

ci_result <- tryCatch({

boot.ci(boot_result, type = "bca")

}, error = function(e) { NULL })

if (!is.null(ci_result) && !is.na(ci_result$bca[1,4])) {

return(list(low = ci_result$bca[1,4], high = ci_result$bca[1,5], method = "BCA"))

} else {

qt <- quantile(boot_result$t, c(0.025, 0.975), na.rm = TRUE)

return(list(low = qt[1], high = qt[2], method = "Percentile"))

}

}

#============ Prepare predicted probabilities for ROC & PR curves ===============

train_prob <- predict(rf_model, train_data, type = "prob")[, "pos"]

train_label <- ifelse(train_data$outcome == "pos", 1, 0)

test_prob <- predict(rf_model, test_data, type = "prob")[, "pos"]

test_label <- ifelse(test_data$outcome == "pos", 1, 0)

\# ========== Training set ROC-AUC + 95%CI ==========

train_roc <- roc(train_label, train_prob, quiet = TRUE)

train_auc_roc <- auc(train_roc)

train_ci_roc <- ci.auc(train_roc, conf.level = 0.95)

\# ========== Test set ROC-AUC + 95%CI ==========

test_roc <- roc(test_label, test_prob, quiet = TRUE)

test_auc_roc <- auc(test_roc)

test_ci_roc <- ci.auc(test_roc, conf.level = 0.95)

\# ========== Training set PR-AUC + 95%CI ==========

train_pr_obj <- pr.curve(scores.class0 = train_prob[train_label == 1],

scores.class1 = train_prob[train_label == 0], curve = TRUE)

train_auc_pr <- train_pr_obj$auc.integral

train_pr_ci <- get_pr_auc_ci(train_prob, train_label)

\# ========== Test set PR-AUC + 95%CI ==========

test_pr_obj <- pr.curve(scores.class0 = test_prob[test_label == 1],

scores.class1 = test_prob[test_label == 0], curve = TRUE)

test_auc_pr <- test_pr_obj$auc.integral

test_pr_ci <- get_pr_auc_ci(test_prob, test_label)

#=============== Console unified output of performance summary ====================

cat("\n==================== Model Performance Summary ====================\n")

cat("【Training set】\n")

cat("ROC-AUC = ", round(train_auc_roc,4),

" 95%CI:", round(train_ci_roc[1],4), "~", round(train_ci_roc[3],4), "\n")

cat("PR-AUC = ", round(train_auc_pr,4),

" 95%CI:", round(train_pr_ci$low,4), "~", round(train_pr_ci$high,4),

" (", train_pr_ci$method, ")\n")

cat("\n【Test set】\n")

cat("ROC-AUC = ", round(test_auc_roc,4),

" 95%CI:", round(test_ci_roc[1],4), "~", round(test_ci_roc[3],4), "\n")

cat("PR-AUC = ", round(test_auc_pr,4),

" 95%CI:", round(test_pr_ci$low,4), "~", round(test_pr_ci$high,4),

" (", test_pr_ci$method, ")\n")

\# ====== Final stable version: SHAP analysis (fastshap package) ===============

cat("\n\n==================== SHAP Analysis ====================\n")

\# 7.2 Prepare dataset for SHAP analysis

\# Remove outcome column, retain features only

train_features <- train_data %>% select(-outcome)

\# fastshap requirement: input matrix/data frame; factors and characters processed uniformly

train_features <- train_features %>%

na.omit() # Remove samples with missing values, consistent with na.action=na.omit in model training

if(nrow(train_features) == 0){

stop("No samples left after removing missing values in train_features, unable to perform SHAP analysis!")

}

\# 7.3 Define prediction function (adapted to rf_model, returns probability of 'pos', consistent with above code)

pred_fun <- function(object, newdata) {

predict(object, newdata = newdata, type = "prob")[, "pos"]

}

\# 7.4 Calculate SHAP values

set.seed(123)

\# Sampling for acceleration; all samples are used when sample size is limited

sample_size <- min(1000, nrow(train_features))

train_features_sample <- train_features[sample(1:nrow(train_features), sample_size), ]

\# Compute SHAP values

shap_values <- fastshap::explain(

object = rf_model,

X = train_features_sample,

pred_wrapper = pred_fun,

nsim = 200, # Number of Monte Carlo samples for SHAP

adjust = TRUE,

parallel = FALSE

)

\# 7.5 SHAP variable importance analysis

\# Method 1: Mean of absolute SHAP values (most commonly used SHAP importance metric in publications)

shap_importance_mean <- apply(shap_values, 2, function(x) mean(abs(x)))

shap_importance_mean_df <- data.frame(

Feature = names(shap_importance_mean),

SHAP_Importance_MeanAbs = shap_importance_mean,

row.names = NULL

) %>% arrange(desc(SHAP_Importance_MeanAbs))

\# Output SHAP variable importance ranking

cat("\n=== SHAP variable importance ranking 1: Mean absolute SHAP value (core metric) ===\n")

print(shap_importance_mean_df)

**Code 4: Code for Random Forest Model Analysis Code Applying SMOTE‑ENN Technique**

library(smotefamily)

library(unbalanced)

library(MASS)

library(rms)

library(pROC)

library(caret)

library(dplyr)

library(tidyr)

library(ResourceSelection)

library(randomForest)

library(PRROC)

library(boot)

\# Basic settings

setwd("C:\\Users\\R\\Desktop\\R\\R")

set.seed(12345)

\# ===================== 1. Data import and preprocessing =====================

data <- read.csv("TP0.csv", fileEncoding = "UTF-8", stringsAsFactors = TRUE)

cat("===== Basic information of raw data =====\n")

cat("Total number of rows: ", nrow(data), "\n")

cat("Number of unique patient IDs: ", length(unique(data[,1])), "\n")

cat("Distribution of outcome variable Y:\n")

orig_table <- table(data$Y)

print(orig_table)

\# Convert specified columns to factors

factor_columns <- c(5, 9, 13, 14,15) # Define column indices to be converted to factors

for (col in factor_columns) {

data[, col] <- as.factor(data[, col])

}

cat("Columns ", paste(factor_columns, collapse = ", "), " have been set as factors.\n")

\# Remove missing values

data <- na.omit(data)

data$Y <- factor(data$Y, levels = c(0, 1), labels = c("0", "1"))

\# 【Revision: Split training/test set by patient ID; no splitting of individual patients】

patient_id_col <- colnames(data)[1]	# The first column contains patient ID

all_patients <- unique(data[[patient_id_col]])

n_pat_total <- length(all_patients)

set.seed(12345)

\# Directly sample patient indices: 70% of patients allocated to training set

train_pat_idx <- sample(1:n_pat_total, size = floor(0.7 * n_pat_total), replace = FALSE)

train_patients <- all_patients[train_pat_idx]

test_patients <- all_patients[-train_pat_idx]

train_data_original <- data[data[[patient_id_col]] %in% train_patients, ]

test_data_original <- data[data[[patient_id_col]] %in% test_patients, ]

cat("\n===== Dataset splitting results by patient ID =====\n")

cat("Total unique patients: ", n_pat_total,"\n")

cat("Patients in training set: ", length(train_patients), "; Sample rows in training set: ", nrow(train_data_original), "\n")

cat("Patients in test set: ", length(test_patients), "; Sample rows in test set: ", nrow(test_data_original), "\n")

\# Error checking

if(length(test_patients)==0 || nrow(test_data_original)==0){

stop("Error: Test set is empty. Please check patient IDs or sampling code!")

}

\# Check class distribution in original training set

cat("\n===== Class distribution of original training set =====\n")

train_table <- table(train_data_original$Y)

print(train_table)

majority_count <- as.numeric(train_table["0"])

minority_count <- as.numeric(train_table["1"])

\# Prepare input data for SMOTE: note that **ID column is removed, ID is not involved in modeling**

X_train <- train_data_original[, !names(train_data_original) %in% c("Y", patient_id_col)]

Y_train <- train_data_original$Y

cat("Converting all non-numeric columns (including factors) to numeric labels before SMOTE...\n")

X_train_for_smote <- X_train

for (col_name in names(X_train_for_smote)) {

if (!is.numeric(X_train_for_smote[[col_name]])) {

X_train_for_smote[[col_name]] <- as.numeric(as.factor(X_train_for_smote[[col_name]])) - 1

cat("Column '", col_name, "' has been converted to numeric.\n", sep="")

}

}

\# ===== 2. SMOTE oversampling (for reference only; ubSMOTE is used in the final model) =====

cat("\n===== SMOTE sampling =====\n")

smote_result <- SMOTE(

X = X_train_for_smote,

target = Y_train,

dup_size = 5,

K = 5

)

train_data_smote <- as.data.frame(smote_result$data)

colnames(train_data_smote)[ncol(train_data_smote)] <- "Y"

train_data_smote$Y <- factor(train_data_smote$Y, levels = c("0", "1"))

cat("===== Results after SMOTE sampling =====\n")

cat("Number of rows after sampling: ", nrow(train_data_smote), "\n")

smote_table <- table(train_data_smote$Y)

print(smote_table)

smote_minority_pct <- round(as.numeric(smote_table["1"])/nrow(train_data_smote)*100, 2)

cat("Proportion of minority class: ", smote_minority_pct, "%\n")

\# ====== 3. ubSMOTE (integrated SMOTE+ENN, final training set) ============

cat("\n===== Implement ubSMOTE (integrated SMOTE+ENN) =====\n")

train_full_for_ubsmote <- cbind(X_train_for_smote, Y = Y_train)

ubsmote_result <- ubSMOTE(

X = train_full_for_ubsmote[, -ncol(train_full_for_ubsmote)],

Y = train_full_for_ubsmote$Y,

perc.over = 300,

perc.under = 150,

k = 3

)

train_data_final <- cbind(ubsmote_result$X, Y = ubsmote_result$Y)

\# ===【Core revision: Rename factor levels to avoid caret errors】======

train_data_final$Y <- factor(train_data_final$Y, levels = c("0", "1"), labels = c("Y0", "Y1"))

cat("\n===== Final ubSMOTE output =====\n")

cat("Total rows in final training set: ", nrow(train_data_final), "\n")

final_table <- table(train_data_final$Y)

print(final_table)

final_minority_pct <- round(as.numeric(final_table["Y1"])/nrow(train_data_final)*100, 2)

cat("Proportion of minority class: ", final_minority_pct, "%\n")

if (length(final_table) < 2) {

stop("ubSMOTE failed to generate balanced data. Check whether sufficient minority samples exist in raw data!")

}

\# = New Module 1: Custom PR-AUC evaluation function + custom random forest for caret grid tuning ==

prSummary <- function(data, lev = NULL, model = NULL) {

pos_level <- lev[2]

prob <- data[, pos_level]

label <- ifelse(data$obs == pos_level, 1, 0)

if(length(unique(label)) < 2){

out <- c(AUCPR = NA)

}else{

pr <- pr.curve(scores.class0 = prob[label == 1], scores.class1 = prob[label == 0], curve = FALSE)

out <- c(AUCPR = pr$auc.integral)

}

names(out) <- "AUCPR"

out

}

\# Custom random forest model supporting simultaneous tuning of mtry + nodesize

rf_custom <- list(

label = "rf_custom",

library = "randomForest",

type = "Classification",

parameters = data.frame(

parameter = c("mtry", "nodesize"),

class = c("numeric", "numeric"),

label = c("mtry", "nodesize")

),

grid = function(x, y, len = NULL, search = "grid") {},

fit = function(x, y, wts, param, lev, last, classProbs, ...) {

randomForest(x = x, y = y,

mtry = param$mtry,

nodesize = param$nodesize,

ntree = 1000,

maxnodes = 1000,

importance = TRUE,

classwt = c(0.5,0.5))

},

predict = function(modelFit, newdata, preProc = NULL, submodels = NULL) {

predict(modelFit, newdata)

},

prob = function(modelFit, newdata, preProc = NULL, submodels = NULL) {

predict(modelFit, newdata, type = "prob")

},

varImp = function(object, ...) {

imp <- importance(object)

out <- data.frame(Overall = imp[,1])

rownames(out) <- rownames(imp)

out

},

levels = function(x) x$classes

)

\# 5-fold cross-validation setup, optimize to maximize AUCPR

ctrl_tune <- trainControl(

method = "cv",

number = 5,

summaryFunction = prSummary,

classProbs = TRUE,

verboseIter = FALSE

)

\# Hyperparameter grid; adjust range as needed

rf_grid <- expand.grid(

mtry = seq(3, 8, by = 1),

nodesize = c(10,15,20,25,30)

)

cat("\n===== Start grid tuning (Objective: maximize AUC-PR) =====\n")

set.seed(12345)

rf_tune <- train(

x = train_data_final[, !colnames(train_data_final) %in% "Y"],

y = train_data_final$Y,

method = rf_custom,

tuneGrid = rf_grid,

trControl = ctrl_tune,

metric = "AUCPR",

maximize = TRUE

)

best_mtry <- rf_tune$bestTune$mtry

best_nodesize <- rf_tune$bestTune$nodesize

cat("Optimal parameters: mtry =", best_mtry, " nodesize =", best_nodesize, "\n")

\# Train final random forest model with optimal hyperparameters

set.seed(12345)

rf_model <- randomForest(

formula = Y ~ .,

data = train_data_final,

ntree = 1000,

mtry = best_mtry,

nodesize = best_nodesize,

maxnodes = 1000,

importance = TRUE,

classwt = c(0.5, 0.5)

)

print(rf_model)

\# ===== New Module 2: Bootstrap internal validation (200 resamples, balanced training set) ====

boot_iter <- 200

boot_metrics <- data.frame(AUCROC = numeric(boot_iter), AUCPR = numeric(boot_iter))

cat("\n===== Start Bootstrap internal validation (", boot_iter, " resamplings) =====\n")

set.seed(12345)

for(b in 1:boot_iter){

boot_idx <- sample(1:nrow(train_data_final), size = nrow(train_data_final), replace = TRUE)

boot_train <- train_data_final[boot_idx, ]

boot_model <- randomForest(

Y ~ .,

data = boot_train,

ntree = 1000,

mtry = best_mtry,

nodesize = best_nodesize,

maxnodes = 1000,

importance = FALSE

)

boot_prob <- predict(boot_model, train_data_final, type = "prob")[,"Y1"]

lab <- ifelse(train_data_final$Y == "Y1", 1, 0)

roc_tmp <- roc(lab, boot_prob, quiet = TRUE)

boot_metrics$AUCROC[b] <- auc(roc_tmp)

pr_tmp <- pr.curve(scores.class0 = boot_prob[lab==1], scores.class1 = boot_prob[lab==0], curve=FALSE)

boot_metrics$AUCPR[b] <- pr_tmp$auc.integral

if(b %% 50 == 0) cat("Completed bootstrap iteration: ", b, "/", boot_iter, "\n")

}

cat("\n===== Summary of Bootstrap internal validation results =====\n")

print(summary(boot_metrics))

cat("Bootstrap mean AUC-ROC =", round(mean(boot_metrics$AUCROC),4),

"95%CI:", round(quantile(boot_metrics$AUCROC,0.025),4), "~", round(quantile(boot_metrics$AUCROC,0.975),4),"\n")

cat("Bootstrap mean AUC-PR =", round(mean(boot_metrics$AUCPR),4),

"95%CI:", round(quantile(boot_metrics$AUCPR,0.025),4), "~", round(quantile(boot_metrics$AUCPR,0.975),4),"\n")

\# ===================== 5. Model prediction =====================

\# Training set prediction (probability of positive class corresponds to "Y1")

train_pred_prob <- predict(rf_model, train_data_final, type = "prob")[, "Y1"]

train_pred_class <- predict(rf_model, train_data_final, type = "class")

\# Test set preprocessing: remove ID column, convert factors to numeric labels

cat("\nPreprocessing test set...\n")

test_data_processed <- test_data_original[, !names(test_data_original) %in% patient_id_col] # Remove ID column

for (col_name in names(test_data_processed)) {

if (!is.numeric(test_data_processed[[col_name]]) & col_name != "Y") {

test_data_processed[[col_name]] <- as.numeric(as.factor(test_data_processed[[col_name]])) - 1

cat("Test set column '", col_name, "' has been converted to numeric.\n", sep="")

}

}

test_pred_prob <- predict(rf_model, test_data_processed, type = "prob")[, "Y1"]

test_pred_class <- predict(rf_model, test_data_processed, type = "class")

\# ===================== 6. Confusion matrix and related analysis =====================

cat("\n===== Confusion matrix and related analysis =====\n")

cat("--- Training set confusion matrix ---\n")

train_cm <- confusionMatrix(train_pred_class, train_data_final$Y, positive = "Y1")

print(train_cm)

cat("\n--- Test set confusion matrix ---\n")

\# Core revision: convert predicted labels Y0/Y1 to 0/1 to match true labels in test set

test_pred_class_01 <- factor(ifelse(test_pred_class == "Y1", "1", "0"), levels = c("0","1"))

test_cm <- confusionMatrix(test_pred_class_01, test_data_processed$Y, positive = "1")

print(test_cm)

cat("\n--- Summary of core evaluation metrics ---\n")

metrics_summary <- data.frame(

Dataset = c("Training set", "Test set"),

Accuracy = c(train_cm$overall["Accuracy"], test_cm$overall["Accuracy"]),

Precision = c(train_cm$byClass["Precision"], test_cm$byClass["Precision"]),

Recall = c(train_cm$byClass["Recall"], test_cm$byClass["Recall"]),

F1_score = c(train_cm$byClass["F1"], test_cm$byClass["F1"])

)

print(metrics_summary)

\# ====== 7. AUC-ROC and AUC-PR (with 95% confidence intervals) =========

cat("\n===== AUC-ROC and AUC-PR (with 95% confidence intervals) =====\n")

calculate_auc_ci <- function(actual, prob, n_boot = 1000, ci_level = 0.95) {

set.seed(12345)

auc_values <- invisible(replicate(n_boot, {

idx <- sample(1:length(actual), replace = TRUE)

roc_obj <- roc(actual[idx], prob[idx], quiet = TRUE)

auc(roc_obj)

}))

ci <- quantile(auc_values, c((1-ci_level)/2, 1-(1-ci_level)/2))

return(list(auc = auc(roc(actual, prob, quiet = TRUE)), ci = ci))

}

calculate_pr_auc_ci <- function(actual, prob, n_boot = 1000, ci_level = 0.95) {

set.seed(12345)

pr_auc_values <- invisible(replicate(n_boot, {

idx <- sample(1:length(actual), replace = TRUE)

pr_obj <- pr.curve(scores.class0 = prob[idx][actual[idx]=="1"],

scores.class1 = prob[idx][actual[idx]=="0"],

curve = FALSE)

pr_obj$auc.integral

}))

pr_auc <- pr.curve(scores.class0 = prob[actual=="1"],

scores.class1 = prob[actual=="0"],

curve = FALSE)$auc.integral

ci <- quantile(pr_auc_values, c((1-ci_level)/2, 1-(1-ci_level)/2))

return(list(pr_auc = pr_auc, ci = ci))

}

\# Convert training set labels to numeric 0/1

train_y_num <- ifelse(train_data_final$Y == "Y1", 1, 0)

cat("--- Training set AUC-ROC ---\n")

train_roc_auc_ci <- calculate_auc_ci(train_y_num, train_pred_prob)

cat(sprintf("AUC-ROC: %.4f (95%% CI: %.4f - %.4f)\n",

train_roc_auc_ci$auc,

train_roc_auc_ci$ci[1],

train_roc_auc_ci$ci[2]))

cat("--- Training set AUC-PR ---\n")

train_pr_auc_ci <- calculate_pr_auc_ci(as.character(train_y_num), train_pred_prob)

cat(sprintf("AUC-PR: %.4f (95%% CI: %.4f - %.4f)\n",

train_pr_auc_ci$pr_auc,

train_pr_auc_ci$ci[1],

train_pr_auc_ci$ci[2]))

cat("\n--- Test set AUC-ROC ---\n")

test_roc_auc_ci <- calculate_auc_ci(as.numeric(as.character(test_data_processed$Y)), test_pred_prob)

cat(sprintf("AUC-ROC: %.4f (95%% CI: %.4f - %.4f)\n",

test_roc_auc_ci$auc,

test_roc_auc_ci$ci[1],

test_roc_auc_ci$ci[2]))

cat("--- Test set AUC-PR ---\n")

test_pr_auc_ci <- calculate_pr_auc_ci(test_data_processed$Y, test_pred_prob)

cat(sprintf("AUC-PR: %.4f (95%% CI: %.4f - %.4f)\n",

test_pr_auc_ci$pr_auc,

test_pr_auc_ci$ci[1],

test_pr_auc_ci$ci[2]))

**Code 5: Code for Restricted Cubic Spline Analysis**

\# Load required libraries

library(rms)	# For fitting restricted cubic spline models

library(ggplot2) # For high‑quality visualization

library(numDeriv) # For numerical derivative calculation

\# --- Data Preparation ---

\# Assume TP2.csv is located under the current working directory

\# Modify setwd() or provide the full file path if needed

setwd("C:/Users/R/Desktop/R/R")

\# Read CSV file with UTF‑8 encoding

data <- read.csv("TP2.csv", fileEncoding = "UTF‑8")

\# Set specified columns (4,5,9,10,14) as factor variables

factor_columns <- c(4, 5, 9, 10, 14)

for(col_idx in factor_columns) {

\# Validate index to avoid errors when CSV contains insufficient columns

if(col_idx <= ncol(data)) {

col_name <- names(data)[col_idx]

data[[col_name]] <- as.factor(data[[col_name]])

cat("Column '", col_name, "' has been converted to factor.\n")

}

}

\# Inspect data structure

cat("Data dimensions:", dim(data), "\n")

cat("Column names:", names(data), "\n")

\# --- Define variables --- Dependent variable Y, main exposure variable P2CO

outcome_var <- "Y"

main_predictor <- "P2CO"

\# Extract names of all remaining variables as covariates to adjust for confounding effects

all_vars <- names(data)

covariates <- all_vars[!all_vars %in% c(outcome_var, main_predictor)]

cat("List of covariates:", paste(covariates, collapse = ", "), "\n")

\# --- Create data distribution object for the rms package ---

\# Critical step for rms; improves model stability

dd <- datadist(data)

options(datadist = "dd")

\# --- Build model formula (reformulate is recommended to avoid warning messages) ---

\# reformulate is the preferred R function for constructing formulas, avoiding string‑concatenation pitfalls

rhs_formula <- c(paste0("rcs(", main_predictor, ", 4)"), covariates)

formula_obj <- reformulate(rhs_formula, response = outcome_var)

\# --- Fit logistic regression model ---

fit <- lrm(formula_obj, data = data, x = TRUE, y = TRUE)

\# --- Inspect model outputs ---

cat("\n=== Model ANOVA Results ===\n")

print(anova(fit))

cat("\n=== Model coefficient summary ===\n")

print(summary(fit))

\# --- Prepare dataset for plotting (using Predict function from rms) ---

\# Create list storing adjusted values for each covariate (mean for numeric variables, mode for categorical variables)

adjust_values_list <- list()

for(cov in covariates) {

if(is.numeric(data[[cov]])) {

adjust_values_list[[cov]] <- mean(data[[cov]], na.rm = TRUE)

} else {

\# Use mode for categorical variables

mode_val <- names(sort(table(data[[cov]]), decreasing = TRUE))[1]

adjust_values_list[[cov]] <- mode_val

}

}

\# do.call safely passes list elements as individual arguments to Predict

args_for_predict <- c(list(

object = fit,

P2CO = seq(min(data[[main_predictor]], na.rm = TRUE),

max(data[[main_predictor]], na.rm = TRUE), length.out = 100),

fun = plogis # Apply inverse logit transformation to obtain predicted probabilities

), adjust_values_list)

\# Call Predict function

pred_obj <- do.call(Predict, args_for_predict)

\# Convert prediction output to data frame for ggplot2

plot_data <- as.data.frame(pred_obj)

\# --- Generate smooth curve with ggplot2 (resolve linewidth warning) ---

p <- ggplot(plot_data, aes(x = P2CO, y = yhat * 100)) +

geom_line(color = "blue", linewidth = 1) + # replace deprecated size with linewidth

geom_ribbon(aes(ymin = lower * 100, ymax = upper * 100), alpha = 0.2, fill = "blue") +

labs(

x = "P2CO",

y = "Treatment success rate (%)",

title = "Adjusted association between P2CO and treatment success rate (Restricted Cubic Spline analysis)"

) +

theme_minimal()

print(p)

\# --- Detect turning points and inflection points on the curve ---

\# 1. Define prediction wrapper function

predict_wrapper <- function(x_val) {

temp_data_row <- data.frame(P2CO = x_val)

for(cov in covariates) {

temp_data_row[[cov]] <- adjust_values_list[[cov]]

}

\# Use fitted instead of type = "fitted" for compatible lrm model invocation

predict(fit, newdata = temp_data_row, type = "fitted")

}

\# 2. Auxiliary function for second‑derivative calculation

second_deriv_func <- function(x) {

grad(function(z) grad(predict_wrapper, z), x)

}

\# 3. Suppress redundant warnings and compute derivatives

cat("\nCalculating first‑ and second‑order derivatives...\n")

plot_data$first_deriv <- suppressWarnings(sapply(plot_data$P2CO, grad, func = predict_wrapper))

plot_data$second_deriv <- suppressWarnings(sapply(plot_data$P2CO, grad, func = second_deriv_func))

\# 4. Remove NA values generated by numerical‑computation errors

plot_data_clean <- plot_data[!is.na(plot_data$first_deriv) & !is.na(plot_data$second_deriv), ]

\# --- Set threshold range to identify extrema and inflection points ---

threshold_lower <- -1e‑3 # Lower threshold

threshold_upper <- 1e‑3	# Upper threshold

\# Extrema: points where first‑derivative lies within threshold range

extreme_points_indices <- which(plot_data_clean$first_deriv >= threshold_lower & plot_data_clean$first_deriv <= threshold_upper)

\# Inflection points: points where second‑derivative lies within threshold range

inflection_points_indices <- which(plot_data_clean$second_deriv >= threshold_lower & plot_data_clean$second_deriv <= threshold_upper)

\# --- Output analytical results ---

cat("\n=== Curve‑analysis outputs ===\n")

\# Output extrema (where first derivative ≈ 0)

if (length(extreme_points_indices) > 0) {

extreme_x <- plot_data_clean$P2CO[extreme_points_indices]

extreme_y <- plot_data_clean$yhat[extreme_points_indices] * 100

cat("Potential extrema detected (first derivative within threshold [", threshold_lower, ", ", threshold_upper, "]):\n")

for (i in 1:length(extreme_x)) {

cat(sprintf(" Extrema: (%.3f, %.2f%%)\n", extreme_x[i], extreme_y[i]))

}

} else {

cat("No extrema detected within observed data range (first derivative within threshold [", threshold_lower, ", ", threshold_upper, "])\n")

}

\# Output inflection points (where second derivative ≈ 0)

if (length(inflection_points_indices) > 0) {

inflection_x <- plot_data_clean$P2CO[inflection_points_indices]

inflection_y <- plot_data_clean$yhat[inflection_points_indices] * 100

cat("Potential inflection points detected (second derivative within threshold [", threshold_lower, ", ", threshold_upper, "]):\n")

for (i in 1:length(inflection_x)) {

cat(sprintf(" Inflection point: (%.3f, %.2f%%)\n", inflection_x[i], inflection_y[i]))

}

} else {

cat("No inflection points detected within observed data range (second derivative within threshold [", threshold_lower, ", ", threshold_upper, "])\n")

}

**Code 6: Code for Venn Diagram Analysis**

library(ggvenn)	# For stable Venn‑diagram plotting

library(ggplot2)

library(STRINGdb)

library(igraph)

library(clusterProfiler)

library(DOSE)

library(pathview)

library(AnnotationDbi)

library(org.Hs.eg.db)

library(enrichplot)

library(ggraph)

setwd("C:/Users/R/Desktop/R/R")

\# Read GeneCards data (female infertility‑related genes)

gene_cards <- dplyr::select(read.csv("GeneCards.csv", stringsAsFactors = FALSE), GeneSymbol, RelevanceScore) %>%

dplyr::rename(gene = GeneSymbol, relevance = RelevanceScore) %>%

dplyr::filter(relevance >= 5) %>%

dplyr::distinct(gene, .keep_all = TRUE)

\# Read PubChem CO target data (human only)

pubchem <- dplyr::select(read.csv("pubchem.csv", stringsAsFactors = FALSE), Gene, Taxonomy_ID) %>%

dplyr::rename(gene = Gene, taxonomy = Taxonomy_ID) %>%

dplyr::filter(taxonomy == "9606") %>%

dplyr::distinct(gene, .keep_all = TRUE)

\# Convert to gene‑symbol vectors

infertility_genes <- gene_cards$gene

co_targets <- pubchem$gene

\# Obtain intersection (unify uppercase format & remove empty entries)

infertility_genes <- toupper(infertility_genes[infertility_genes != ""])

co_targets <- toupper(co_targets[co_targets != ""])

intersection_genes <- intersect(infertility_genes, co_targets)

\# Export intersecting gene list (for subsequent enrichment analysis)

write.csv(data.frame(Intersection_Genes = intersection_genes),

"intersection_genes.csv", row.names = FALSE)

print(paste("Number of intersecting genes:", length(intersection_genes)))

print(head(intersection_genes))

\# ===================== Core modification: Plot Venn diagram =====================

\# Construct input list for Venn plotting

venn_data <- list(

"Female Infertility" = infertility_genes,

"CO Targets" = co_targets

)

\# Generate Venn plot

p <- ggvenn(venn_data,

fill_color = c("skyblue", "salmon"),

fill_alpha = 0.5,

stroke_size = 0.5,

text_size = 5,

set_name_size = 5)

\# Save output as PDF file

ggsave("venn_diagram.pdf", p, width = 6, height = 6, device = "pdf")

\# ==================================================================

print("Venn diagram has been saved successfully")
