# Supplementary material for: Effect of air pollution on clinical pregnancy outcomes following intrauterine insemination: a machine learning-based analysis
Source: Front Public Health. 2026 Sep 18;14:1931648. doi: 10.3389/fpubh.2026.1931648 (PMC13630761; doi:10.3389/fpubh.2026.1931648)
Supplement: Supplementary file 2 [file Table_1.docx]

**Table S1.** The assigned value in generalized estimating equations analysis

| **Variable** | **Assigned value** |
| --- | --- |
| Female age (y) |  |
| Age＜30 | 0 |
| 30≤Age＜35 | 1 |
| Age≥35 | 2 |
|  |  |
| Male age (y) |  |
| Age＜30 | 0 |
| 30≤Age＜35 | 1 |
| Age≥35 | 2 |
|  |  |
| basal FSH-to-LH ratio |  |
| Ratio＜2 | 0 |
| 2≤Ratio＜3 | 1 |
| Ratio≥3 | 2 |
|  |  |
| Type of infertility |  |
| Primary | 0 |
| Secondary | 1 |
|  |  |
| Endometrial thickness (mm) |  |
| Thickness＜8 | 0 |
| 8≤Thickness＜10 | 1 |
| 10≤Thickness＜12 | 2 |
| Thickness≥12 | 3 |
|  |  |
| ≥14mm follicles(n) |  |
| 1 | 0 |
| ≥2 | 1 |
|  |  |
| Total progressive motile sperm count after semen processing (×10^6^) |  |
| Total count＜10 | 0 |
| 10≤Total count＜20 | 1 |
| 20≤Total count＜30 | 2 |
| Total count≥30 | 3 |
|  |  |
| Luteal phase support |  |
| No | 0 |
| Yes | 1 |
|  |  |
| Stimulation protocol |  |
| Natural cycle | 0 |
| Ovulation inducing cycle | 1 |
|  |  |
| Infertility duration (y) |  |
| Years＜3 | 0 |
| 3≤Years＜5 | 1 |
| Years≥5 | 2 |
|  |  |
| Female BMI (kg/ m^2^) |  |
| BMI＜18.5 | 0 |
| 18.5≤BMI＜25 | 1 |
| BMI≥25 | 2 |
|  |  |
| Season |  |
| Spring | 0 |
| Summer | 1 |
| Autumn | 2 |
| Winter | 3 |
|  |  |
| Calendar year |  |
| 2014-2015 | 0 |
| 2016-2017 | 1 |
| 2018-2019 | 2 |
| 2020-2021 | 3 |
| 2022-2023 | 4 |
| 2024-2025 | 5 |
|  |  |
| Number of IUI cycles |  |
| 1 | 0 |
| 2 | 1 |
| ≥3 | 2 |
|  |  |
| Clinical pregnancy |  |
| No | 0 |
| Yes | 1 |
|  |  |
| PM_2.5_ in Period 1（μg/m^3^） |  |
| PM_2.5_ ＜17.21 | 0 |
| 17.21 ≤ PM_2.5_＜24.55 | 1 |
| 24.55 ≤ PM_2.5_＜33.93 | 2 |
| PM_2.5_ ≥ 33.93 | 3 |
|  |  |
| PM_10_ in Period 1（μg/m^3^） |  |
| PM_10_ ＜29.51 | 0 |
| 29.51 ≤ PM_10_＜40.03 | 1 |
| 40.03 ≤ PM_10_＜51.11 | 2 |
| PM_10_≥ 51.11 | 3 |
|  |  |
| SO_2_ in Period 1（μg/m^3^） |  |
| SO_2_ ＜5.11 | 0 |
| 5.11 ≤ SO_2_＜6.71 | 1 |
| 6.71 ≤ SO_2_＜10.17 | 2 |
| SO_2_≥ 10.17 | 3 |
|  |  |
| NO_2_ in Period 1（μg/m^3^） |  |
| NO_2_ ＜20.15 | 0 |
| 20.15 ≤ NO_2_＜26.94 | 1 |
| 26.94 ≤ NO_2_＜39.32 | 2 |
| NO_2_≥ 39.32 | 3 |
|  |  |
| O_3_ in Period 1（μg/m^3^） |  |
| O_3_ ＜66.71 | 0 |
| 66.71≤ O_3_＜81.80 | 1 |
| 81.80 ≤ O_3_＜95.23 | 2 |
| O_3_ ≥ 95.23 | 3 |
|  |  |
| CO in Period 1（mg/m^3^） |  |
| CO ＜0.57 | 0 |
| 0.57 ≤ CO ＜0.74 | 1 |
| 0.74 ≤ CO＜0.99 | 2 |
| CO ≥ 0.99 | 3 |
|  |  |
| PM_2.5_ in Period 2（μg/m^3^） |  |
| PM_2.5_ ＜15.43 | 0 |
| 15.43 ≤ PM_2.5_＜23.64 | 1 |
| 23.64 ≤ PM_2.5_＜33.80 | 2 |
| PM_2.5_ ≥ 33.80 | 3 |
|  |  |
| PM_10_ in Period 2（μg/m^3^） |  |
| PM_10_ ＜27.14 | 0 |
| 27.14 ≤ PM_10_＜37.79 | 1 |
| 37.79 ≤ PM_10_＜51.71 | 2 |
| PM_10_≥ 51.71 | 3 |
|  |  |
| SO_2_ in Period 2（μg/m^3^） |  |
| SO_2_ ＜5.00 | 0 |
| 5.00 ≤ SO_2_＜6.79 | 1 |
| 6.79 ≤ SO_2_＜9.71 | 2 |
| SO_2_≥ 9.71 | 3 |
|  |  |
| NO_2_ in Period 2（μg/m^3^） |  |
| NO_2_ ＜18.27 | 0 |
| 18.27 ≤ NO_2_＜26.57 | 1 |
| 26.57 ≤ NO_2_＜39.86 | 2 |
| NO_2_≥ 39.86 | 3 |
|  |  |
| O_3_ in Period 2（μg/m^3^） |  |
| O_3_ ＜58.29 | 0 |
| 58.29≤ O_3_＜79.21 | 1 |
| 79.21 ≤ O_3_＜103.38 | 2 |
| O_3_ ≥ 103.38 | 3 |
|  |  |
| CO in Period 2（mg/m^3^） |  |
| CO ＜0.56 | 0 |
| 0.56 ≤ CO ＜0.73 | 1 |
| 0.73 ≤ CO＜0.95 | 2 |
| CO ≥ 0.95 | 3 |
|  |  |

Abbreviations: BMI, body mass index; FSH, follicle-stimulating hormone; LH, luteinizing hormone.
