# Supplementary material for: Effect of air pollution on clinical pregnancy outcomes following intrauterine insemination: a machine learning-based analysis
Source: Front Public Health. 2026 Sep 18;14:1931648. doi: 10.3389/fpubh.2026.1931648 (PMC13630761; doi:10.3389/fpubh.2026.1931648)
Supplement: Supplementary file 3 [file Table_2.docx]

**Table S2 Spearman correlation coefficients of six air pollutants during two periods**

| Periods | Pollutants | PM_2.5_ | PM_10_ | SO_2_ | NO_2_ | O_3_ | CO |
| --- | --- | --- | --- | --- | --- | --- | --- |
| Period 1 | PM_2.5_ | 1 | 0.966 | 0.666 | 0.963 | -0.073 | 0.752 |
|  | PM_10_ |  | 1 | 0.6286 | 0.941 | -0.022 | 0.677 |
|  | SO_2_ |  |  | 1 | 0.648 | -0.250 | 0.752 |
|  | NO_2_ |  |  |  | 1 | -0.102 | 0.743 |
|  | O_3_ |  |  |  |  | 1 | -0.404 |
|  | CO |  |  |  |  |  | 1 |
|  |  |  |  |  |  |  |  |
| Period 2 | PM_2.5_ | 1 | 0.963 | 0.650 | 0.909 | 0.143 | 0.697 |
|  | PM_10_ |  | 1 | 0.625 | 0.891 | 0.168 | 0.636 |
|  | SO_2_ |  |  | 1 | 0.630 | -0.105 | 0.676 |
|  | NO_2_ |  |  |  | 1 | 0.022 | 0.695 |
|  | O_3_ |  |  |  |  | 1 | -0.264 |
|  | CO |  |  |  |  |  | 1 |

**Table S3 VIFs under alternative model specifications during two periods**

| Period | Model Specification | PM_2.5_ | PM_10_ | SO_2_ | NO_2_ | O_3_ | CO |
| --- | --- | --- | --- | --- | --- | --- | --- |
| Period 1 | Model 1: All 6 pollutants | 26.34 | 17.70 | 3.05 | 9.40 | 1.45 | 4.59 |
|  | Model 2: Excl. PM_2.5_ | —— | 8.33 | 3.05 | 6.78 | 1.44 | 4.25 |
|  | Model 3: Excl. PM_10_ | 12.39 | —— | 2.87 | 9.32 | 1.44 | 4.52 |
|  | Model 4: Excl. PM_2.5_ & PM₁₀ | —— | —— | 2.66 | 2.29 | 1.34 | 4.19 |
|  |  |  |  |  |  |  |  |
| Period 2 | Model 1: All 6 pollutants | 16.21 | 12.29 | 2.32 | 5.65 | 1.41 | 3.05 |
|  | Model 2: Excl. PM_2.5_ | —— | 5.90 | 2.32 | 4.47 | 1.36 | 2.77 |
|  | Model 3: Excl. PM_10_ | 7.78 | —— | 2.20 | 5.54 | 1.39 | 3.03 |
|  | Model 4: Excl. PM_2.5_ & PM₁₀ | —— | —— | 2.09 | 1.90 | 1.14 | 2.64 |

**Table S4 The exposure distributions of the six air pollutants during two periods**

| Period | Pollutant | Mean | SD | Min | P25. Q1 | Median | P75. Q3 | Max | IQR |
| --- | --- | --- | --- | --- | --- | --- | --- | --- | --- |
| Period 1 | PM_2.5_（μg/m^3^） | 26.75 | 12.59 | 6.66 | 17.21 | 24.55 | 33.93 | 73.58 | 16.72 |
|  | PM_10_（μg/m^3^） | 41.77 | 15.89 | 17.66 | 29.51 | 40.03 | 51.11 | 109.45 | 21.60 |
|  | SO_2_（μg/m^3^） | 8.58 | 5.52 | 2.84 | 5.11 | 6.71 | 10.17 | 40.83 | 5.07 |
|  | NO_2_（μg/m^3^） | 30.17 | 13.12 | 8.74 | 20.15 | 26.94 | 39.32 | 70.44 | 19.17 |
|  | O_3_（μg/m^3^） | 81.51 | 23.35 | 33.22 | 66.71 | 81.80 | 95.23 | 179.97 | 28.52 |
|  | CO（mg/m^3^） | 0.80 | 0.30 | 0.35 | 0.57 | 0.74 | 0.99 | 1.69 | 0.42 |
|  |  |  |  |  |  |  |  |  |  |
| Period 2 | PM_2.5_（μg/m^3^） | 26.61 | 14.83 | 4.79 | 15.43 | 23.64 | 33.80 | 102.93 | 18.38 |
|  | PM_10_（μg/m^3^） | 41.50 | 19.04 | 11.93 | 27.14 | 37.79 | 51.71 | 144.36 | 24.57 |
|  | SO_2_（μg/m^3^） | 8.37 | 5.86 | 1.86 | 5.00 | 6.79 | 9.71 | 57.07 | 4.71 |
|  | NO_2_（μg/m^3^） | 30.36 | 15.95 | 6.64 | 18.27 | 26.57 | 39.86 | 93.79 | 21.59 |
|  | O_3_（μg/m^3^） | 82.58 | 33.36 | 11.79 | 58.29 | 79.21 | 103.38 | 245.00 | 45.09 |
|  | CO（mg/m^3^） | 0.80 | 0.32 | 0.27 | 0.56 | 0.73 | 0.95 | 1.96 | 0.39 |

Abbreviations: IQR, interquartile range; SD, standard deviation.

**Table S5 GEE analysis of different pollutant models during Period 1**

| Period | Model | Pollutant | aOR | 95% CI | | P-value |
| --- | --- | --- | --- | --- | --- | --- |
|  |  |  |  | Lower | Upper |  |
| Period 1 | single-pollutant | PM_2.5_ | 1.135 | 0.926 | 1.391 | 0.224 |
|  |  | PM_10_ | 1.090 | 0.897 | 1.324 | 0.386 |
|  |  | SO_2_ | 0.999 | 0.799 | 1.25 | 0.994 |
|  |  | NO_2_ | 1.051 | 0.869 | 1.272 | 0.609 |
|  |  | O_3_ | 1.107 | 0.966 | 1.268 | 0.144 |
|  |  | CO | 1.063 | 0.794 | 1.422 | 0.681 |
|  |  |  |  |  |  |  |
|  | two-pollutant | PM_2.5_ | 1.133 | 0.917 | 1.402 | 0.248 |
|  |  | CO | 1.005 | 0.744 | 1.358 | 0.974 |
|  |  |  |  |  |  |  |
|  |  | PM_10_ | 1.084 | 0.886 | 1.326 | 0.431 |
|  |  | CO | 1.028 | 0.762 | 1.388 | 0.855 |
|  |  |  |  |  |  |  |
|  |  | SO_2_ | 0.995 | 0.795 | 1.246 | 0.966 |
|  |  | CO | 1.064 | 0.794 | 1.424 | 0.679 |
|  |  |  |  |  |  |  |
|  |  | NO_2_ | 1.041 | 0.850 | 1.274 | 0.699 |
|  |  | CO | 1.044 | 0.767 | 1.421 | 0.784 |
|  |  |  |  |  |  |  |
|  |  | O_3_ | 1.110 | 0.968 | 1.274 | 0.135 |
|  |  | CO | 1.082 | 0.806 | 1.453 | 0.601 |
|  |  |  |  |  |  |  |
|  |  | PM_2.5_ | 1.099 | 0.887 | 1.361 | 0.389 |
|  |  | O_3_ | 1.088 | 0.943 | 1.256 | 0.246 |
|  |  |  |  |  |  |  |
|  |  | PM_10_ | 1.043 | 0.844 | 1.289 | 0.696 |
|  |  | O_3_ | 1.095 | 0.945 | 1.270 | 0.229 |
|  |  |  |  |  |  |  |
|  |  | SO_2_ | 0.985 | 0.788 | 1.232 | 0.894 |
|  |  | O_3_ | 1.108 | 0.966 | 1.270 | 0.142 |
|  |  |  |  |  |  |  |
|  |  | NO_2_ | 1.027 | 0.845 | 1.247 | 0.791 |
|  |  | O_3_ | 1.103 | 0.961 | 1.267 | 0.162 |
|  |  |  |  |  |  |  |
|  | multi-pollutant | SO_2_ | 0.976 | 0.779 | 1.224 | 0.835 |
|  |  | NO_2_ | 1.013 | 0.823 | 1.249 | 0.900 |
|  |  | O_3_ | 1.11 | 0.964 | 1.279 | 0.148 |
|  |  | CO | 1.079 | 0.786 | 1.480 | 0.639 |

Abbreviations: GEE, generalized estimating equation.

**Table S6 GEE analysis of different pollutant models during Period 2**

| Period | Model | Pollutant | aOR | 95% CI | | P-value |
| --- | --- | --- | --- | --- | --- | --- |
|  |  |  |  | Lower | Upper |  |
| Period 2 | single-pollutant | PM_2.5_ | 1.125 | 0.960 | 1.320 | 0.146 |
|  |  | PM_10_ | 1.147 | 0.981 | 1.342 | 0.085 |
|  |  | SO_2_ | 0.992 | 0.829 | 1.187 | 0.929 |
|  |  | NO_2_ | 1.119 | 0.929 | 1.347 | 0.236 |
|  |  | O_3_ | 1.119 | 0.995 | 1.26 | 0.061 |
|  |  | CO | 0.789 | 0.617 | 1.010 | 0.059 |
|  |  |  |  |  |  |  |
|  | two-pollutant | PM_2.5_ | 1.257 | 1.052 | 1.502 | 0.012 |
|  |  | CO | 0.689 | 0.526 | 0.904 | 0.007 |
|  |  |  |  |  |  |  |
|  |  | PM_10_ | 1.251 | 1.057 | 1.480 | 0.009 |
|  |  | CO | 0.703 | 0.541 | 0.914 | 0.009 |
|  |  |  |  |  |  |  |
|  |  | SO_2_ | 1.010 | 0.842 | 1.210 | 0.917 |
|  |  | CO | 0.788 | 0.616 | 1.009 | 0.059 |
|  |  |  |  |  |  |  |
|  |  | NO_2_ | 1.252 | 1.022 | 1.533 | 0.030 |
|  |  | CO | 0.698 | 0.534 | 0.911 | 0.008 |
|  |  |  |  |  |  |  |
|  |  | O_3_ | 1.123 | 0.997 | 1.264 | 0.057 |
|  |  | CO | 0.786 | 0.613 | 1.007 | 0.057 |
|  |  |  |  |  |  |  |
|  |  | PM_2.5_ | 1.069 | 0.900 | 1.271 | 0.445 |
|  |  | O_3_ | 1.097 | 0.966 | 1.247 | 0.155 |
|  |  |  |  |  |  |  |
|  |  | PM_10_ | 1.092 | 0.913 | 1.306 | 0.334 |
|  |  | O_3_ | 1.086 | 0.950 | 1.243 | 0.228 |
|  |  |  |  |  |  |  |
|  |  | SO_2_ | 0.972 | 0.813 | 1.163 | 0.758 |
|  |  | O_3_ | 1.122 | 0.997 | 1.263 | 0.056 |
|  |  |  |  |  |  |  |
|  |  | NO_2_ | 1.080 | 0.892 | 1.308 | 0.429 |
|  |  | O_3_ | 1.105 | 0.978 | 1.249 | 0.109 |
|  |  |  |  |  |  |  |
|  | multi-pollutant | SO_2_ | 0.953 | 0.794 | 1.143 | 0.601 |
|  |  | NO_2_ | 1.222 | 0.990 | 1.507 | 0.062 |
|  |  | O_3_ | 1.090 | 0.963 | 1.234 | 0.171 |
|  |  | CO | 0.709 | 0.543 | 0.927 | 0.012 |

Abbreviations: GEE, generalized estimating equation.

**Table S7 Sensitivity analysis of pollutants during Period 2**

| Variable | Regression coefficients |
| --- | --- |
| PM_2.5_ | 0 |
| PM_10_ | 0 |
| SO_2_ | 0 |
| NO_2_ | 0 |
| O_3_ | 0.0035 |
| CO | -0.2059 |
